# Biomineralization-inspired Crystallization of Manganese Oxide on Silk Fibroin Nanoparticles for *in vivo* MR/fluorescence Imaging-assisted Tri-modal Therapy of Cancer

**DOI:** 10.7150/thno.36252

**Published:** 2019-08-14

**Authors:** Ruihao Yang, Mengmeng Hou, Ya Gao, Shiyu Lu, Lei Zhang, Zhigang Xu, Chang Ming Li, Yuejun Kang, Peng Xue

**Affiliations:** 1 Key Laboratory of Luminescent and Real-Time Analytical Chemistry (Southwest University), Ministry of Education, School of Materials and Energy, Southwest University, Chongqing 400715, China.; 2 Chongqing Engineering Research Center for Micro-Nano Biomedical Materials and Devices, Southwest University, Chongqing 400715, China.; 3 Institute of Sericulture and Systems Biology, Southwest University, Chongqing 400716, China.

**Keywords:** Silk fibroin, Tumor microenvironment, Combination therapy, MR/fluorescence imaging, Manganese Oxide.

## Abstract

Regenerated silk fibroin (SF) is a type of natural biomacromolecules with outstanding biocompatibility and biodegradability. However, stimulus-responsive SF-based nanocomplex has seldom been reported for application in tumor diagnosis and therapy.

**Methods**: As a proof-of-concept study, a multifunctional SF@MnO_2_ nanoparticle-based platform was strategically synthesized using SF as a reductant and a template *via* a biomineralization-inspired crystallization process in an extremely facile way. Because of their mesoporous structure and abundant amino and carboxyl terminal residues, SF@MnO_2_ nanoparticles were co-loaded with a photodynamic agent indocyanine green (ICG) and a chemotherapeutic drug doxorubicin (DOX) to form a SF@MnO_2_/ICG/DOX (SMID) nanocomplex.

**Results**: The obtained product was highly reactive with endogenous hydrogen peroxide (H_2_O_2_) in tumor microenvironment, which was decomposed into O_2_ to enhance tumor-specific photodynamic therapy (PDT). Moreover, SMID nanocomplex produced a strong and stable photothermal effect upon near-infrared (NIR) irradiation for photothermal therapy (PTT) owing to the distinct photothermal response of SF@MnO_2_ and stably conjugated ICG*.* The concurrent NIR fluorescence and magnetic resonance (MR) imaging *in vivo* both indicated effective tumor-specific enrichment of SMID nanoparticles *via* enhanced permeability and retention (EPR) effect. Animal studies further verified that SMID nanoparticles remarkably improved tumor inhibitive efficacy through combination PTT/PDT/chemotherapy with minimal systemic toxicity or adverse effect.

**Conclusion**: This study demonstrated the promising potential of SF-based nanomaterial to address some of the key challenges in cancer therapy due to unfavorable tumor microenvironment for drug delivery.

## Introduction

Nanoscale drug delivery systems (NDDS), aiming to deliver diagnostic or therapeutic agents into site of lesion while reducing systemic toxicity, have received increasing attention in the field of nanomedicine during the past few decades 1-4. Particularly, imaging-guided theranostic platforms for simultaneous detection and eradication of tumors have become tremendously attractive owing to their efficacy, controllability and spatial selectivity 5-7. As a typical carrier material, inorganic nanoparticles have been extensively exploited for imaging-guided therapy, including metal (Au), metal chalcogenide (Bi_2_Se_3_, WO_2.9_, CuS), semiconductor nanocrystals (quantum dots) and lanthanide-doped upconversion nanoparticles (UCNPs) 8-12. However, it is very difficult to incorporate functional therapeutic molecules into these inorganic nanocarriers owing to the weak binding affinity or lack of active chemical ligands on the surface of carrier materials 13, 14, which is usually mitigated by sophisticated and costly synthetic procedures. Moreover, multiple stimuli-responsive drug-loaded nanoparticles are demanded to realize spatiotemporal control of drug release with higher tumor-targeting performance and ameliorated adverse effects to normal tissues 15, 16. Therefore, a rapid and economical synthetic strategy is of great importance to develop drug-loaded inorganic nanoparticles as novel imaging contrast agents and multifunctional therapeutic drugs for cancer treatment, taking advantage of various exogenous or endogenous stimuli.

Regenerated silk fibroin (SF) is a natural protein originated from the cocoon of silkworm *Bombyx mori*, which contains both hydrophilic and hydrophobic domains with a large amount of reactive amino and carboxyl groups in SF peptide 17, 18. Many biological and chemical modifications can be facilely implemented to conjugate functional components with the peptide, thereby introducing unique properties to the SF-based nanomaterials. Given the remarkable mechanical strength, biocompatibility, biodegradability and flexibility of peptide modification, SF-derived biomaterials have been widely applied in biomedical and pharmaceutical fields in forms of nanoparticles, films, scaffolds, hydrogels, fibers, et al 19-21. In particular, SF-based nanoparticles have extremely low toxicity and definite metabolic pathways 22, 23. Moreover, SF nanoparticles have a large specific surface area and can be homogeneously dispersed in aqueous media, acting as a template or a reducing agent for surface functionalization or catalytic reactions 24, 25. Previous reports indicated that biomacromolecules were closely related to bioinspired mineralization process. For instance, the mineralization of CaCO_3_ can be induced and regulated by SF-based nanostructures through an oriented crystallization process 26, 27. Therefore, controllable growth of inorganic substances on the skeleton of SF could be achieved by biomineralization-inspired metal reduction, rendering new possibilities of therapeutic-related functionalization of SF matrix. These characteristics are particularly attractive in drug delivery applications that are aimed to achieve efficient loading of multiple theranostic agents, controlled drug release responsive to disease-specific endogenous or exogenous stimuli, and combination multimodal therapeutics.

Tumor hypoxia is caused by the rapidly proliferating cancer cells enclosed in the distorted tumor vasculatures, which severely hinders the treatment efficacy of photodynamic therapy (PDT) owing to the insufficient oxygen supply in tumor microenvironment 28-30. To date, various supplementary strategies have been applied to relieve tumor hypoxia and facilitate conventional PDT, such as by promoting intratumoral blood flow, delivering oxygen locally *via* tumor-targeted gas shuttles, or producing oxygen *in situ* from endogenous H_2_O_2_ that are catalyzed by natural or nano-enzymes 31-36. Notably, MnO_2_ nanoparticles can induce enzymatic decomposition of H_2_O_2_ into water and oxygen, and thus may work as a critical mediator to reduce tumor hypoxia 34, 35. Moreover, MnO_2_ can decompose in response to other tumor-specific environmental cues, such as glutathione (GSH) or local acidity, and the produced Mn^2+^ is an excellent contrast agent for T1-magnetic resonance (MR) imaging of tumoral tissues 37, 38. In addition to tumor hypoxia, there are other challenges that restrain the efficacy of conventional PDT, such as inefficient accumulation or heterogeneous distribution of systemically injected photosensitizers in tumoral tissues 39. Fortunately, based on the passive or active tumor-targeting property of nanocarriers, photosensitizers can be delivered on-target with enhanced permeation and bioavailability at tumor sites by regulating the size and surface charge of carriers 40-42. Therefore, it is of significant interest to develop photosensitizer-loaded functional nanostructures responsive to tumor-specific microenvironment, aiming to realize highly efficient tumor accumulation and relieve tumor hypoxia for an enhanced PDT.

**Figure 1:**
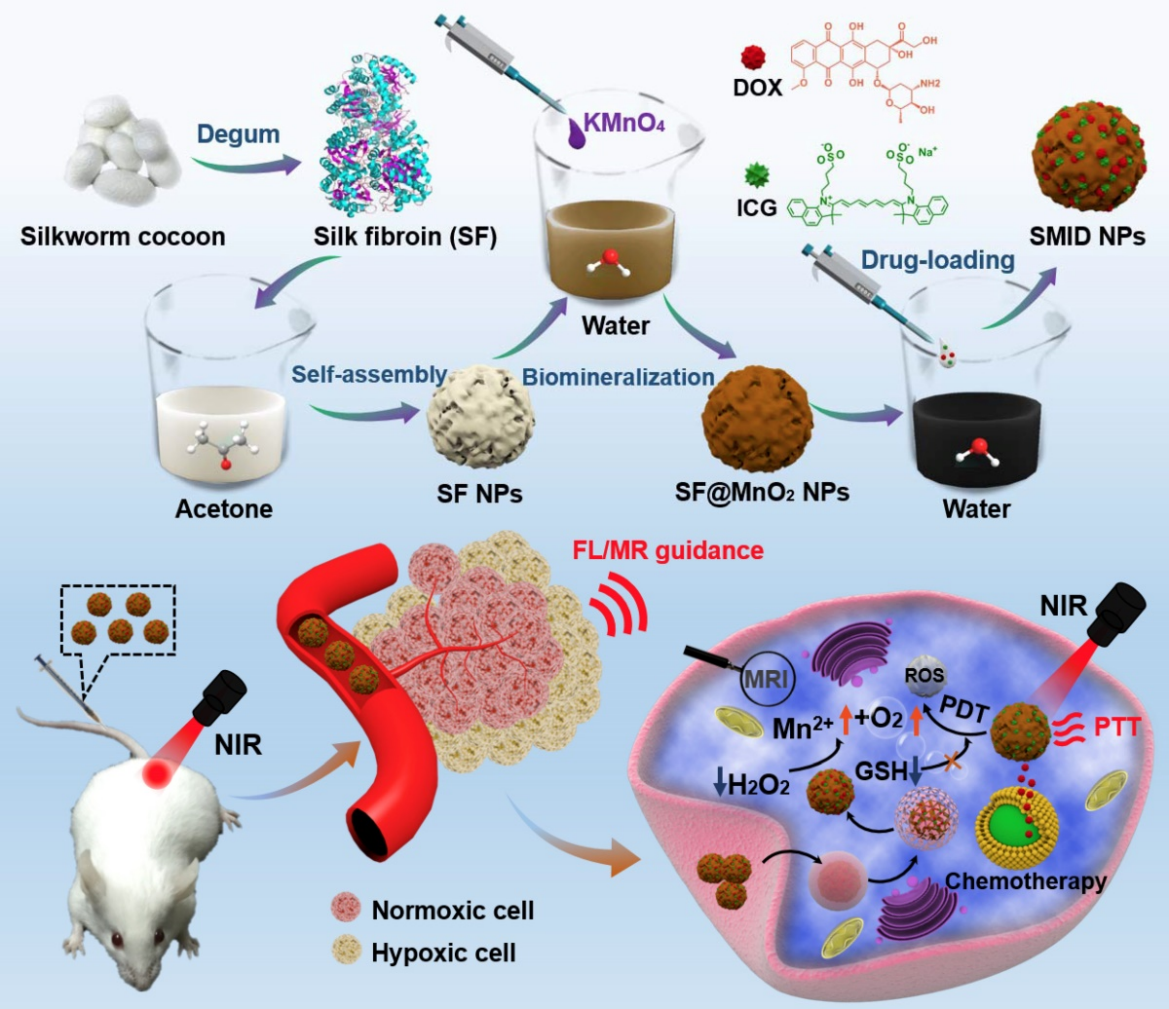
Schematic of the synthetic procedure of SF@MnO_2_/ICG/DOX (SMID) nanoparticles as a multifunctional drug delivery platform for *in vivo* MR/fluorescence imaging-assisted tri-modal therapy of cancer.

In this study, clinically approved photosensitizer indocyanine green (ICG) and chemotherapeutic drug doxorubicin (DOX) were co-immobilized into SF nanoparticles, where the particle surface was mineralized by MnO_2_ through a bioinspired crystallization process (Figure 1). As a proof-of-concept, we developed a novel strategy to engineer the SF@MnO_2_ nanoparticles in an extremely facile means based on one-pot fabrication technique, mimicking the disinfection process of KMnO_4_. SF nanoparticles served as a template as well as a reducing agent during the reduction process. Then, ICG was encapsulated into SF@MnO_2_ nanoparticles under the interaction between the pigment and side chains of amino acid residues in SF 43. Meanwhile, DOX was loaded into SF@MnO_2_ nanoparticles through the active carboxyl group residues on SF to realize stimuli-responsive drug delivery 44. Hypothetically, the obtained SF@MnO_2_/ICG/DOX (SMID) nanoparticles are able to accumulate in tumor region *via* the enhanced permeability and retention (EPR) effect after intravenous injection. After cell internalization through endocytosis, the MnO_2_ component can react with the prevalent cancer cell metabolite H_2_O_2_ and produce abundant O_2_ for PDT. The reaction product Mn^2+^ is a contrast agent for concurrent high performance T_1_-weighted MRI. The encapsulated ICG plays a dual role as a potent photosensitizer and a photothermal adjuvant to SF@MnO_2_-mediated hyperthermia *in situ*. Additionally, the highly fluorescent ICG molecules make it possible for fluorescence visualization of tumors *in vivo* 45, 46. Finally, SMID-mediated DOX release is pH-dependent, which is favorable in the acidic tumor microenvironment. In this way, the multifunctional SMID nanoparticles may realize MR/fluorescence dual-modal imaging-guided combination PDT/PTT/chemotherapy. The physicochemical properties and anti-tumor performance of this theranostic nanoplatform was characterized and verified systematically *in vitro* using tumor cell lines and *in vivo* based on tumor-bearing mouse models.

## Results and Discussion

### Preparation and characterizations of SMID nanoparticles

The synthetic procedure of SMID nanoparticles is illustrated in Figure 1. Silk fibers (bave) comprise two individual SF filaments (brins) that are covered by a glue-like protein silk sericin. Silk sericin is believed to cause many problems, such as poor biocompatibility, hypersensitivity and inflammatory reactions, which is unacceptable for biomedical applications 47. Therefore, flocculent SF proteins was usually isolated from natural *bombyx mori* cocoons by hydrolyzing the intermediate bonds of sericin in a standard degumming process (Figure S1). Subsequently, SF nanoparticles were prepared by organic solvent-induced self-assembly of SF chains. The attenuation of non-covalent interactions and cleavage of disulfide bonds usually result in unfolding of SF chains into a random coil/helix structure. In the presence of acetone, the conformation of SF chain changed from a linear structure to β-sheets, resulting in the self-assembly of SF into nanoparticles. SF@MnO_2_ nanoparticles were synthesized based on one-step bioinspired crystallization, mimicking the disinfection process using KMnO_4_. During this process, SF served as a reductant as well as a template, and the reduction product MnO_2_ was mineralized *in situ* following the redox reaction between KMnO_4_ and SF. Afterwards, ICG/DOX complex were conjugated with SF@MnO_2_ nanostructure through the interaction between the drug molecules and the active amino residuals on SF.

**Figure 2:**
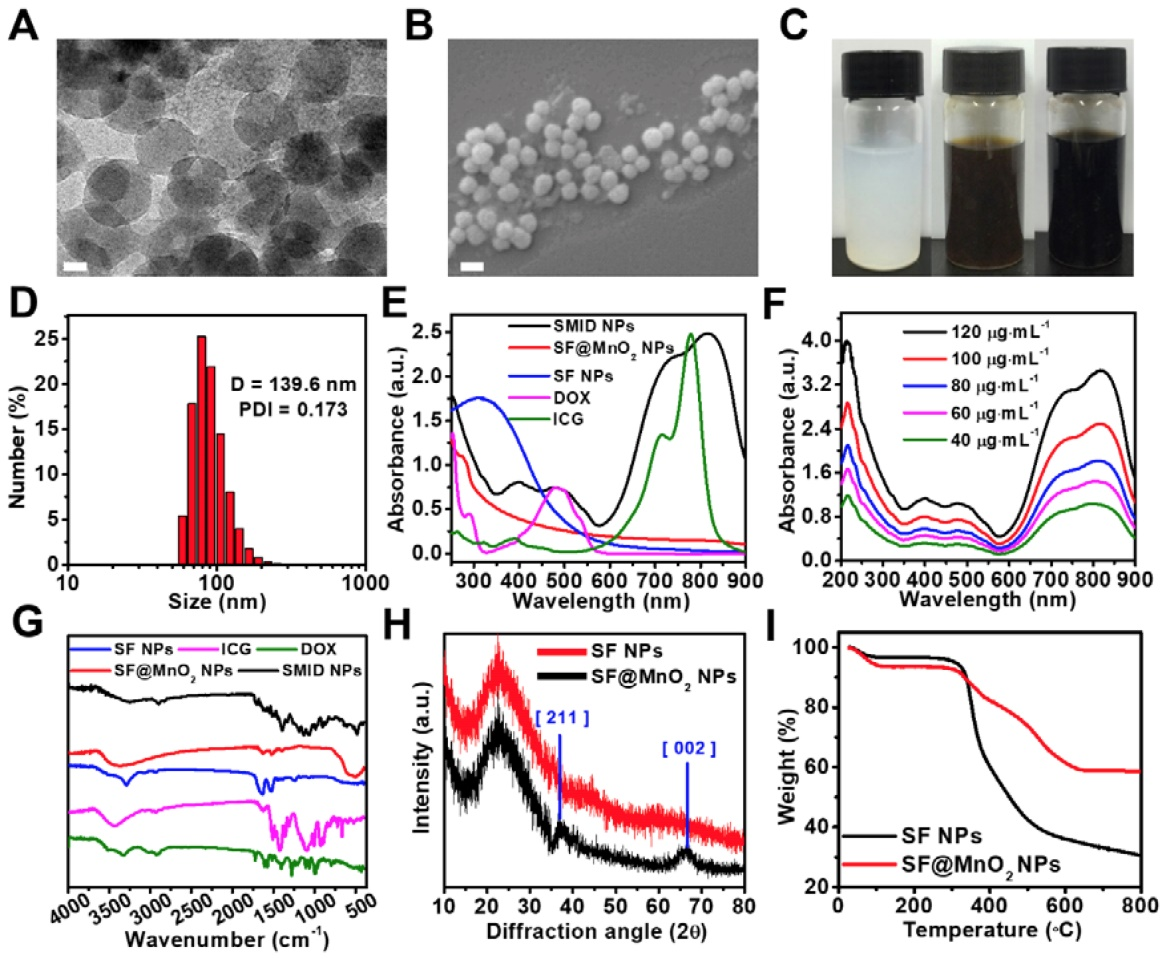
Physicochemical characterizations of SMID nanoparticles: (A) TEM image of SMID nanoparticles (scale bar: 20 nm); (B) FESEM image of SMID nanoparticles (scale bar: 100 nm); (C) images of the aqueous dispersions of SF, SF@MnO_2_ and SMID nanoparticles (from left to right); (D) hydrodynamic size distribution of SMID nanoparticles measured by DLS; (E) UV-vis-NIR absorption spectra of ICG, DOX and the dispersions of SF, SF@MnO_2_ and SMID nanoparticles; (F) UV-vis-NIR absorption spectra of SMID nanoparticles under various concentrations; (G) FT-IR spectra of ICG, DOX and the dispersions of SF, SF@MnO_2_ and SMID nanoparticles; (H) XRD patterns of SF and SF@MnO_2_ nanoparticles; (I) TGA curves of SF and SF@MnO_2_ nanoparticles.

The morphology of as-prepared SF, SF@MnO_2_ and SMID nanoparticles was characterized by TEM and SEM. As shown in Figure S2A and S2c, the obtained SF nanoparticles exhibited a well-defined spheroid structure with an average size of 42.46 ± 7.91 nm. After *in situ* growth of MnO_2_, the diameter of nanoparticles increased to 61.9 ± 1.5 nm (Figure S2B and S2D). The final SMID nanoparticles exhibited a similar sphere-like shape with a diameter of 60.1 ± 2.8 nm (Figure 2A-B). Meanwhile, the hydrodynamic sizes and polydispersity index (PDI) of SF, SF@MnO_2_ and SMID nanoparticles were measured as 140.3 nm (PDI = 0.166), 201 nm (PDI = 0.207) and 139.6 nm (PDI = 0.173), respectively (Figure 2D, S3). Compared to the dehydrated size measured by TEM, a much greater hydrodynamic diameter as measured by DLS was attributed to the swelling of SF and potential clustering effect of nanoparticles in aqueous suspension 48, 49. It was interesting that the hydrodynamic size of SMID nanoparticles became considerably smaller than that of bare carrier SF@MnO_2_ after drug loading. As reported previously, the cross-linking density of SF scaffold is inversely correlated to its degree of swelling 23. Therefore, we speculate that the active carboxyl groups and side chains of amino acid residues in SF may interact with the primary amine and sulfonate groups in drug molecules during drug loading, resulting in a higher cross-linking density of SF matrix and hence the smaller hydrodynamic size of SMID nanoparticles. The decreased zeta potential of SMID nanoparticles after drug loading implied an enhanced colloidal stability, which could be attributed to the effective conjugation of positive charged ICG/DOX complex (Figure S4). The SMID nanoparticles showed negligible size variation for 7 days in PBS, DMEM or FBS (10%) solutions, indicating their long-term stability (Figure S5). Compared to the milk white color of SF nanoparticle dispersion, the vivid dark brown color of SF@MnO_2_ nanoparticle dispersion suggested the successful reduction of KMnO_4_ *in situ* (Figure 2C). Notably, the aqueous dispersion of SMID nanoparticles exhibited an aterrimus color.

The Raman spectra of SF and SF@MnO_2_ were recorded to analyze their chemical compositions (Figure S6). Raman scattering bands at 1600-1700 cm^-1^ and 1200-1300 cm^-1^ respectively assigned to amide I and amide III were found in both SF and SF@MnO_2_ nanoparticles, indicating the existence of SF molecular structure 50. However, SF@MnO_2_ also showed a characteristic broad peak in the range of 550-650 cm^-1^, corresponding to the symmetric (Mn-O) stretching vibration of MnO_6_ octahedra 51. These results suggested that SF nanoparticles were partially oxidized during preparation of SF@MnO_2_ nanoparticles and crystalline MnO_2_ was formed on the SF substrate. To understand the underlying mechanism of the bioinspired mineralization of MnO_2_ on SF, the reaction time was extended to 1 h. The spherical structure of SF@MnO_2_ nanoparticles started to collapse or deform as compared to the products obtained previously after 15 min of reaction (Figure S7). After the reaction was further extended to 24 h, SF nanoparticles completely vanished while only single-crystal nanosheets of MnO_2_ could be observed (Figure S8). These findings suggested that SF was gradually consumed as a reductant when the reaction continued. In other words, higher amount of mineralized MnO_2_ *in situ* could unavoidably alter the sphere-like morphology of SF nanoparticles.

The UV-vis-NIR absorption spectrum of SMID nanoparticles was measured to analyze their formation (Figure 2E). Three characteristic peaks at ~488 nm, ~730 nm and ~820 nm indicated the successful loading of DOX and ICG into the SF@MnO_2_ matrix. In comparison with the characteristic absorbance peaks of free ICG at 710 nm and 785 nm, these redshifted peaks of ICG after drug loading could be ascribed to the formation of ICG dimers and oligomers, also reported as J-aggregates 52. The resultant SMID nanoparticles exhibited strong light absorbing capability in NIR region with an absorbance intensity positively correlated to the sample concentration, implying a good potential as a PTT agent (Figure 2F). FT-IR spectrometry of the key components (DOX, ICG, SF and SF@MnO_2_ nanoparticles) were applied to confirm the composition of SMID nanoparticles. The peaks at 1651 cm^-1^ and 1511 cm^-1^ of SF were attributed to the amide I band and amide II band of protein, and the peak at 3289 cm^-1^ revealed the presence of abundant hydroxyl groups 53. The peak at ~490 cm^-1^ of SF@MnO_2_ indicated a typical Mn-O bond vibrations in MnO_2_ 54. Meanwhile, the broad peaks at 900-1100 cm^-1^ and 1400-1500 cm^-1^ of ICG molecules were ascribed to the vinyl stretches and C=C stretches, respectively 55. Typical peaks of DOX were present at 3450 cm^-1^ and 3325 cm^-1^ owing to the stretching vibrations of N-H and O-H groups, respectively 56. Therefore, the FT-IR spectrum of SMID nanoparticles included the characteristic peaks of all the building blocks, verifying the successful synthesis of final products. In contrast to SF nanoparticles, the XRD pattern of SF@MnO_2_ (Figure 2H) displayed two distinct peaks at 2θ = 36.64° and 65.52°, corresponding to the characteristic peaks of α-MnO_2_ (JCPDS card 44-0141). Additionally, high resolution TEM images of SF@MnO_2_ nanoparticles showed a lattice spacing of 0.24 nm, which agreed well with the (2 1 1) crystal planes of tetragonal MnO_2_ (Figure S9) 57. Such interplanar spacing was in accordance with that measured from XRD pattern. The less weight loss of SF@MnO_2_ than SF nanoparticles at a terminal temperature of 800^o^C further demonstrated an effective MnO_2_ mineralization on the SF matrix (Figure 2I).

**Figure 3:**
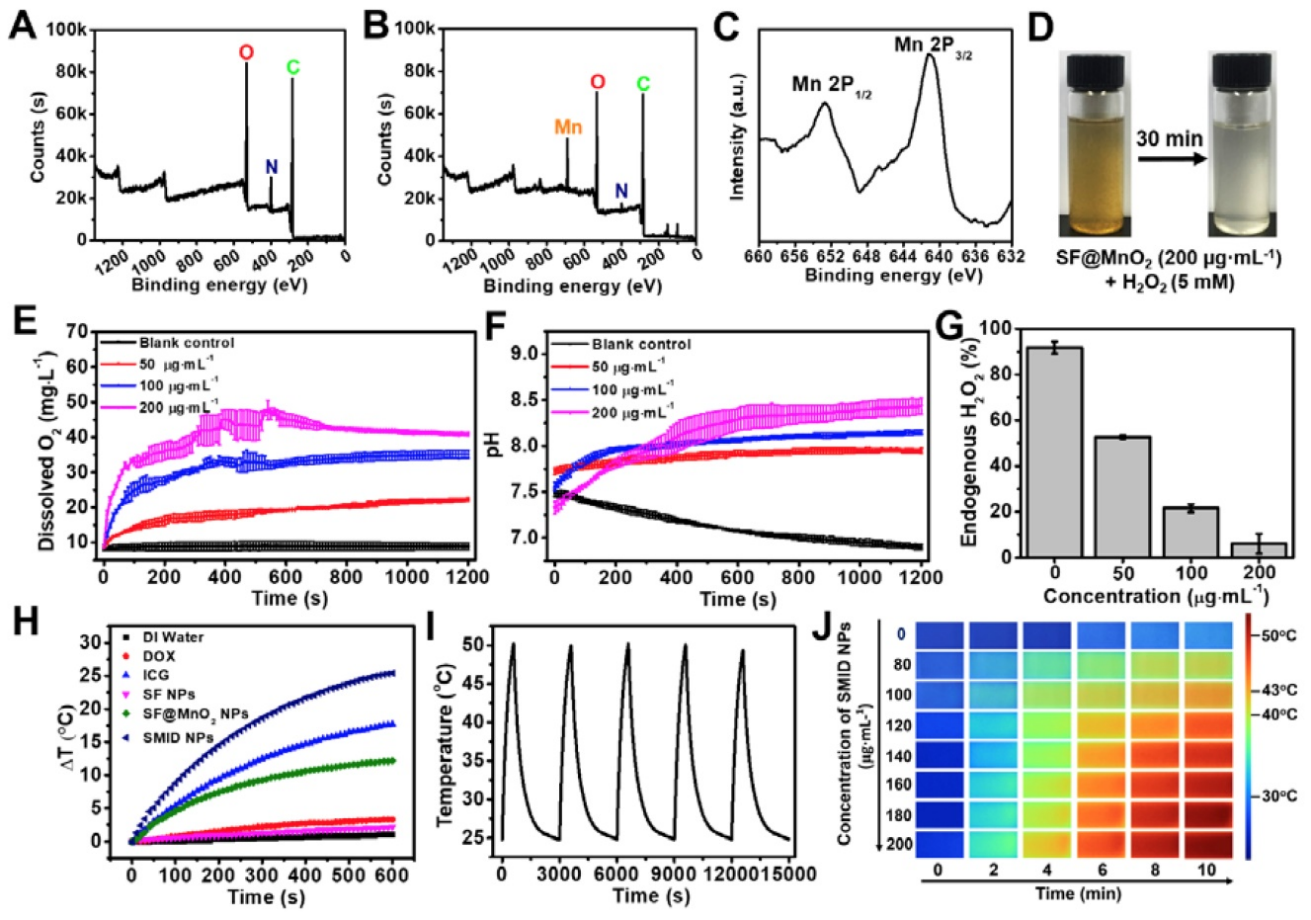
XPS characterizations including full survey spectra of (A) SF and (B) SF@MnO_2_ nanoparticles, and a core level spectrum of (C) Mn2p; (D) images of the mixture of SF@MnO_2_ (200 µg·mL^-1^) and H_2_O_2_ (5 mM) before and after 30 min of incubation; (E) simultaneous O_2_ generation and (F) pH variation of H_2_O_2_ solution (1 mM) after adding SF@MnO_2_ nanoparticles with different concentrations; (G) quenching of endogenous level of H_2_O_2_ (1 mM) by SF@MnO_2_ nanoparticles with different concentrations for 20 min; (H) temperature elevation of the aqueous dispersions of various agents (equivalent ICG concentrations: 16 µg·mL^-1^); (I) temperature change of SMID nanoparticle dispersion (equivalent ICG concentrations: 16 µg·mL^-1^) under on/off laser irradiations for 5 cycles (laser on for 10 min per cycle); (J) thermographic images of the cuvettes containing SMID nanoparticle dispersion with various concentrations subject to laser irradiation for up to 10 min.

The valence states and chemical composition of SMID nanoparticles were further analyzed by XPS. Compared to the XPS spectrum of SF nanoparticles, a typical peak of Mn emerged and the signal intensity of N element decreased after bioinspired mineralization, clearly indicating a redox reaction between the oxidizing KMnO_4_ and the reducing amidogen group of protein (Figure 3A-B). More specifically, two peaks centered at 641.8 and 652.9 eV in the core level XPS spectrum could be assigned to Mn2p_3/2_ and Mn2p_1/2_ of MnO_2_, respectively (Figure 3C). There were several other minor peaks in the spectrum of SF@MnO_2_ at ~100 eV (Si2p), ~160 eV (S2p) and ~830 eV (unknown), which could be attributed to other externally introduced elements during sampling or reaction process. In contrast, these minor peaks were absent in SF spectrum probably due to the shielding effect of SF molecular structure, similar to the findings reported previously by Sim et al 58. Moreover, energy dispersive spectroscopy (EDS) spectrum of SF@MnO_2_ nanoparticles further confirmed the existence of Mn element, demonstrating the successful deposition of MnO_2_ on SF (Figure S10) 59. The BET surface area of SF@MnO_2_ nanoparticles was measured as 66.213 m^2^·g^-1^ with an average pore size of 2.054 nm (Figure S11). The highly porous structure of the obtained SF@MnO_2_ is highly advantageous for high-dosage drug loading.

### *In vitro* H_2_O_2_ decomposition capacity

MnO_2_ is known as an excellent inorganic catalyst that can trigger the decomposition of H_2_O_2_ into H_2_O and O_2_ to ameliorate the hypoxia and improve the treatment efficacy of PDT. The catalysis includes the following reactions.

2MnO_2_ + H_2_O_2_ = 2MnOOH + O_2_ (1)

2MnOOH + 4H^+^ + H_2_O_2_ = 2Mn^2+^ + 4H_2_O + O_2_ (2)

2MnOOH + 2H^+^ = MnO_2_ + Mn^2+^ + 2H_2_O (3)

We firstly evaluated the feasibility of whether SF@MnO_2_ nanoparticles could render O_2_ generation *in vitro* by reacting with H_2_O_2_. As shown in Figure 3D, a large number of tiny bubbles were clearly observed immediately after mixing of SF@MnO_2_ (200 µg·mL^-1^) and H_2_O_2_ (5 mM). Meanwhile, the color fading from brown to white implied the consumption of MnO_2_ during 30 min of catalytic reactions. Additionally, more rapid generation of O_2_ could be triggered by higher concentration of SF@MnO_2_ nanoparticles, and the amount of dissolved O_2_ also increased with longer reaction time (Figure 3E). It was noted that the dissolved O_2_ level reached saturation after about 10 min of reaction under the SMID concentration of 200 µg·mL^-1^. The pH values in various reaction systems were concurrently monitored (Figure 3F). The decreasing pH in the blank control group could be due to the electrolytic dissociation of H_2_O_2_. In contrast, pH value slightly increased in the groups treated with SMID nanoparticles, owing to the consumption of H^+^ ions and the production of the intermediate product MnOOH during the catalytic reactions 60. In a quantitative study, SMID nanoparticles (200 µg·mL^-1^) were found to quench 85.7% of endogenous H_2_O_2_ (1 mM) in 20 min (Figure 3G). Alternatively, DCFH-DA assay also revealed a remarkable intracellular H_2_O_2_ consumption owing to the catalase-like activity of SF@MnO_2_ (SM) and SMI nanoparticles (Figure S12).

### Photothermal effect and stability

The photothermal properties of SMID nanoparticles were evaluated by exposing the sample-laden cuvette (equivalent ICG concentration: 16 µg·mL^-1^) to an NIR laser (808 nm, 2 W·cm^-2^), and the temperature variation was monitored using a digital thermometer (Figure 3H). SF@MnO_2_ and ICG showed a temperature increase of 12.2^o^C and 17.7^o^C during 10 min of irradiation, respectively, which were considerably higher than the control groups of DI water, DOX and SF. The remarkable photothermal response of SF@MnO_2_ carrier suggested that it could serve as a novel PTT agent for tumor therapy. Meanwhile, the temperature elevation of SMID nanoparticles was 25.4^o^C, attributed to the additive photothermal effect of SF@MnO_2_ carrier and the photosensitizer ICG. Moreover, the temperature increase of the SMID dispersion was dependent on both agent concentration and laser output power density (Figure S13A-B), implying a good opportunity to modulate the photothermal performance of this nanoplatform by adjusting these two parameters accordingly. Under repetitive NIR irradiations, SMID nanoparticle dispersion showed a notable photostability with only 3.15% decrease in the peak temperature after five cycles of irradiation, which was much better than that of free ICG with 17.15% reduction in the peak temperature (Figure 3I, S13C). As reported in the literature, silk fibroin or serum albumin prevented the interaction between encapsulated ICG and the surrounding environment, thereby decreasing the decomposition while improving the photothermal and colloidal stability of ICG 43, 61. Similarly, the SF@MnO_2_ carrier in the present study provided an effective shield to stabilize the encapsulated ICG molecules and also contributed to the strong photothermal response itself, resulting in a considerably high photothermal conversion efficiency (η) of 35.65% (Figure S14).

### Stimuli-responsive drug release *in vitro*

**Figure 4:**
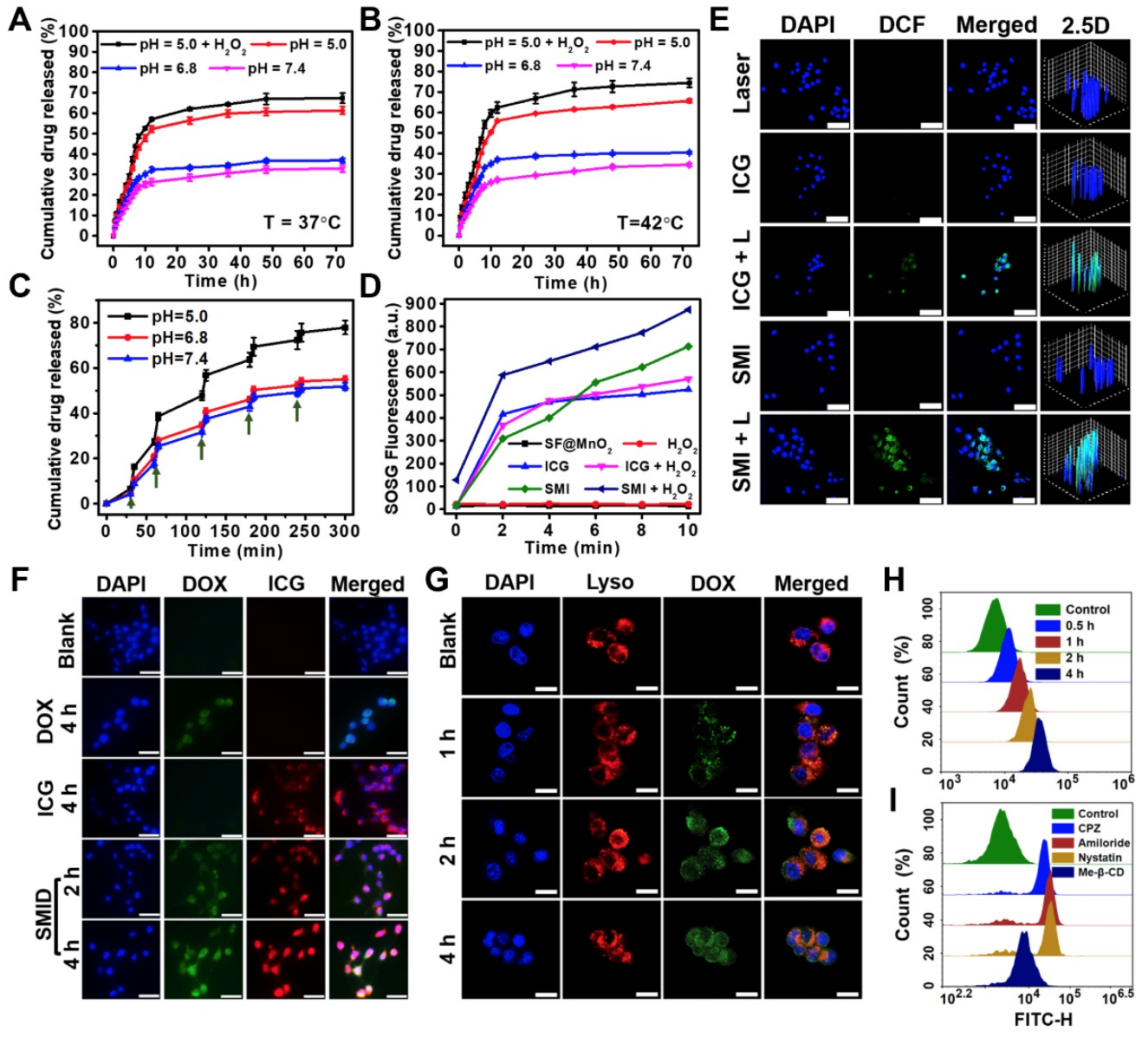
Release kinetics of DOX from SMID nanoparticles under various pH conditions at (A) 37^o^C, (B) 42^o^C or (C) 37^o^C with periodic laser irradiations (green arrows indicate the initiation of laser irradiation for 5 min; H_2_O_2_ concentration: 100 µM); (D) generation of ^1^O_2_ determined by SOSG fluorescence probe under NIR laser irradiation (808 nm, 2 W·cm^-2^) for 10 min (H_2_O_2_ concentration: 100 µM); (E) detection of intracellular ROS generation using DCFH-DA probe (FITC channel) after various treatments, where “L” stands for NIR irradiation for 10 min (scale bars: 50 µm); (F) fluorescence imaging of 4T1 cells after treatment with DOX (green), ICG (red) and SMID (equivalent ICG concentration: 10 μg·mL^-1^) for various period of time (scale bars: 25 µm); (G) confocal images showing the subcellular localization of SMID nanoparticles after various incubation time (red and green colors denote the fluorescence of lysosomes and DOX, respectively; scale bars: 25 µm); flow cytometry analysis of cellular uptake of SMID nanoparticles in 4T1 cells (H) after various co-incubation time or (I) treated with different endocytosis inhibitors for 4 h.

The drug loading performance of SF for DOX and ICG were investigated by mixing the SF nanoparticles and drug solutions at room temperature. A stable and homogeneously colored dispersion were obtained after centrifugation and washing for three times, suggesting the good affinity between the carrier and drugs (Figure S15). As revealed in a previous study, the coupling of DOX with ICG originates from the weak electrostatic interaction between the primary amine group in DOX and the sulfonate group in ICG during immobilization, which favors the high loading content on drug carriers 62. To optimize the drug loading content (DLC) and encapsulation efficiency (EE) for both drugs, different mass ratios (2:1, 1:1 and 1:2) of DOX to ICG were assessed in the co-loading process. The highest EE for DOX and ICG were achieved as 96.13% and 90.75%, respectively, under the mass ratio of DOX: ICG = 1:1. The optimal DLC for DOX and ICG was obtained as 16.69% and 8.13%, respectively, under the same drug feeding ratio (Figure S16). DOX release kinetics of SMID nanoparticles was studied under the effects of different endogenous and exogenous stimuli, including pH, temperature and NIR laser irradiation. During 72 h of incubation at pH 7.4, only 32.9% and 34.6% of DOX were released from SMID nanoparticles at 37^o^C and 42^o^C, respectively, indicating a decent stability of drug encapsulation under the mimic physiological condition (Figure 4A-B). In contrast, accelerated DOX release occurred in acidic solution (pH = 6.8 and 5) at both temperatures, owing to the acid-triggered decomposition of MnO_2_ and protonation of amino group in DOX. Moreover, higher concentration of H_2_O_2_ induced a greater amount of DOX release from SMID nanoparticles, which could be due to the consumption of MnO_2_ and further exposure of more DOX molecules to the surrounding medium (Figure S17). Notably, the presence of H_2_O_2_ significantly accelerated drug release at pH 5.0, which could be ascribed to the expedited decomposition of MnO_2_ in the catalytic reactions as shown in formula (1)-(3). In addition, the drug release rate was slightly higher under 42^o^C than in 37^o^C, which was in accordance with a previous finding that temperature increase could induce the disruption of ICG/DOX complex and thus enhance the solubility of DOX 62. Furthermore, the drug release showed a highly responsive behavior to NIR light exposures, which tremendously accelerated drug release in each of the pulsatile irradiation cycles (Figure 4C). These findings provided solid evidence that drug release from SMID nanoparticles could be precisely regulated by NIR irradiation and environmental acidity.

### Enhanced ROS production

PDT depends on photosensitizers that convert oxygen from ground state (triplet state) to cytotoxic singlet oxygen (^1^O_2_) under light activation and thus induce apoptosis of cancer cells. Considering the capability of SF@MnO_2_ nanoparticles in producing O_2_ in the presence of H_2_O_2_ as shown above, we speculated that ICG-conjugated SF@MnO_2_ (SMI) could mediate an enhanced photodynamic yield of ^1^O_2_ in a tumor-specific H_2_O_2_-rich environment. Therefore, we measured the production of ^1^O_2_ mediated by SF@MnO_2_, ICG or SMI under laser irradiation (808 nm, 2 W·cm^-2^, 10 min) based on the recovered fluorescence of SOSG probe in the presence of ^1^O_2_ (Figure 4D). As expected, SMI nanoparticles exhibited much higher rate of ^1^O_2_ yield than free ICG, which could be due to the enhanced stability of ICG in form of J-aggregates after conjugation with nanocarriers. More importantly, significantly increased ^1^O_2_ production by SMI nanoparticles was observed in the presence of H_2_O_2_, following the MnO_2_-catalyzed O_2_ supply after H_2_O_2_ decomposition. SMI-mediated ^1^O_2_ yield was also evaluated using a DPBF probe, and the obtained results were similar to those using SOSG probe (Figure S18). Furthermore, intracellular ^1^O_2_ yield *in situ* induced by photosensitizers was analyzed using a DCFH-DA probe, which can be oxidized by ROS to generate DCF with green fluorescence (Figure 4E). There was no DCF fluorescence observed in the groups free from laser irradiation, indicating negligible ROS levels in their cytoplasm. Meanwhile, ICG- or SMI-treated groups showed clear fluorescence in DCF channel after NIR laser irradiation. Compared to the cells treated with free ICG, treatment with SMI nanoparticles induced a substantially higher ROS level, which agreed well with the above findings of ^1^O_2_ yield in solution (Figure 4D). Moreover, we demonstrated the intracellular yield of ^1^O_2_ using SOSG. As expected, the most outstanding ^1^O_2_ yield was found in the group of “SMI + H_2_O_2_” upon laser irradiation as proved by the strongest green fluorescence among all groups, which was due to the amelioration of hypoxia after SMI-mediated decomposition of H_2_O_2_ into O_2_ (Figure S19).

On the other hand, the higher GSH level in tumor cells relative to normal cells may result in partly consumption of ROS, compromising the treatment efficacy of PDT 38. Meanwhile, the level of intracellular GSH can be reduced by MnO_2_ through a redox reaction 63, in which GSH is oxidized into glutathione disulfide (GSSG) *via* thiol-disulfide exchange while MnO_2_ is reduced to Mn^2+^ as specified in the following reaction.

MnO_2_ + 2 GSH + 2 H^+^ → Mn^2+^ + GSSG + 2 H_2_O (4)

As expected, SF@MnO_2_ nanoparticles exhibited a GSH depletion effect depending on the nanoparticle concentrations (Figure S20A). Further experiments on tumor cells also showed a similar capability of SF@MnO_2_ in depleting intracellular GSH (Figure S20B). Therefore, the GSH-induced consumption of ROS could be minimized by the SF@MnO_2_ carrier, while more ROS could be reserved to facilitate SMID-mediated PDT.

### Cellular uptake

Cellular uptake of SMID nanoparticles was studied *in vitro* using fluorescence microscopy (Figure 4F). Firstly, 4T1 cells were incubated with DOX, ICG and SMID nanoparticles (equivalent ICG concentration: 10 μg·mL^-1^) for different periods of time. The cells incubated with SMID nanoparticles showed quite strong fluorescence signals from both DOX and ICG channels, whereas those treated with free drugs showed much lower fluorescence intensity most probably due to the limited cellular uptake efficiency. Cell internalization of SMID nanoparticles was visualized after staining with LysoTracker Red (Figure 4G). Initially, increasing DOX fluorescence in 4T1 cells showed gradual overlap with the signals of LysoTracker over the incubation time up to 2 h, suggesting the endocytosis of these nanoparticles transported by lysosomes. However, such fluorescence overlap became weakened after 4 h, implying that DOX was released and diffused from lysosomes to cytoplasm. Because lysosomes are membrane-bound acidic vesicles (pH: 4.5-5.0), DOX release could be accelerated in this acidic environment as demonstrated above *in vitro* (Figure 4A). Meanwhile, local H^+^ were consumed in the reactions with MnO_2_, which prevented further acidification of lysosomal vesicles while triggered ATPase-mediated influx of protons and counter ions, resulting in the osmotic swelling and rupture of lysosome membrane, or previously reported “proton-sponge” effect 64. We used flow cytometry analysis to evaluate the nanoparticle uptake efficiency based on large cell populations. The increasing peak intensity of DOX fluorescence over time in single cells indicated the time-dependent cellular uptake of nanoparticles, which reached a remarkable cellular uptaking rate of 99.77% in 4 h (Figure 4H, S21). To further understand the specific endocytotic mechanism, 4T1 cells were subject to various endocytosis inhibitors during incubation with SMID nanoparticles. As displayed in Figure 4I, the cells pretreated with Me-β-CD (lipid-raft-mediated) exhibited the most significant suppression on the peak fluorescence intensity compared to those treated with other endocytosis inhibitors, including CPZ (clathrin-mediated), amiloride (macropinocytosis-mediated) and nystatin (caveolar-mediated). Moreover, cellular uptake of SMID nanoparticles was obviously suppressed at 4^o^C or in the presence of NaN_3_ (inhibitor of ATP hydrolase) as shown in Figure S22. Therefore, it could be inferred that cell internalization of SMID nanoparticles was an energy-dependent process *via* the lipid-raft-mediated endocytosis pathway.

### *In vitro* cytotoxicity

**Figure 5:**
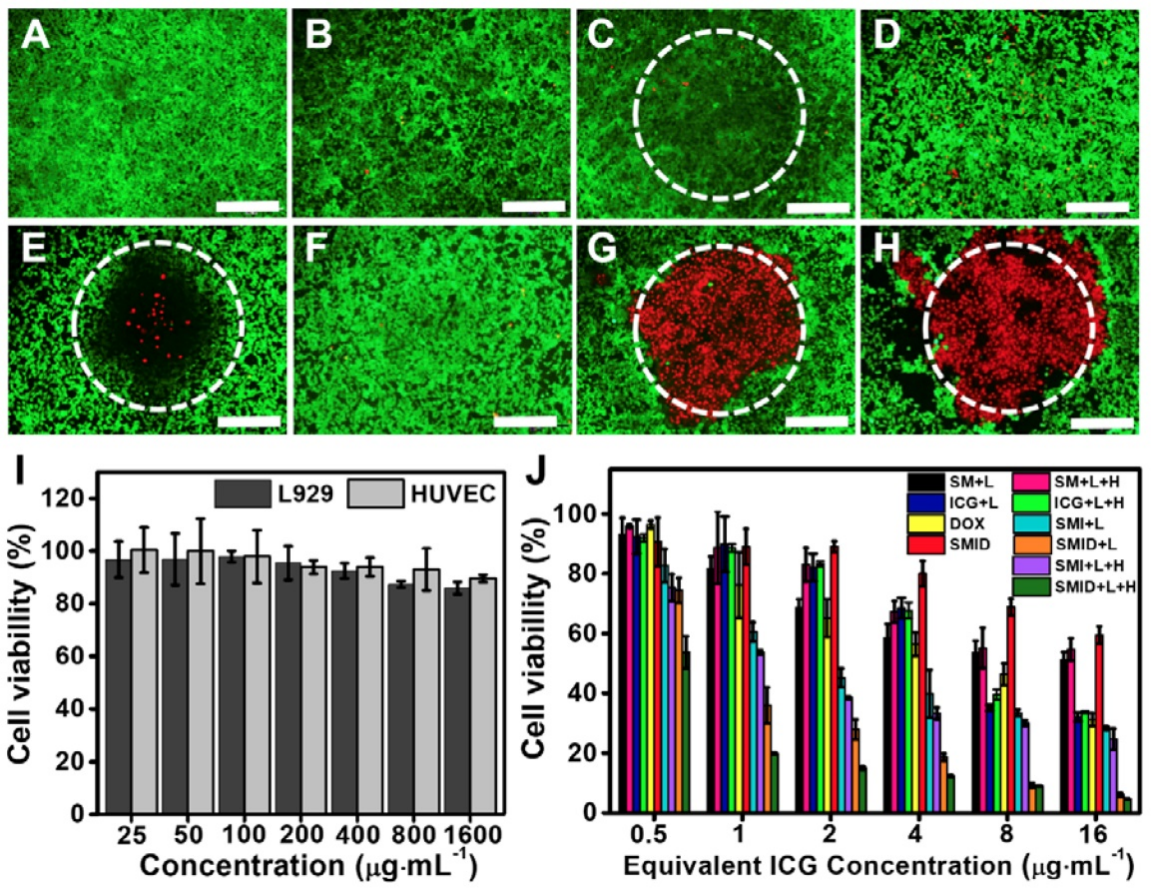
Fluorescence images of 4T1 cells stained by a Live/Dead viability/cytotoxicity kit after various treatments, including (A) blank control, (B) H_2_O_2_, (C) NIR laser, (D) DOX; (E) ICG plus laser, (F) SMID, (G) SMID plus laser, (H) SMID plus laser and H_2_O_2_ (equivalent ICG concentration: 16 µg·mL^-1^; H_2_O_2_: 100 µM; laser irradiation: 5 min). The dashed circles denote the laser irradiation spots (scale bars: 200 µm). The green and red dots represent live and dead cells, respectively. (I) Viability of HUVECs and L929 cells after treatment with SF@MnO_2_ nanoparticles at various concentrations for 24 h. (J) Viability of 4T1 cells after various treatments (“L” indicates the NIR laser irradiation for 5 min, “H” denotes H_2_O_2_ at 100 µM).

The biocompatibility of SF@MnO_2_ nanocarrier was investigated using human umbilical vein endothelial cells (HUVECs) and L929 mouse fibroblasts by standard MTT assays. The cell viability was found to remain above 80% after 24 h even under the carrier concentration as high as 1600 µg·mL^-1^, indicating the extremely low cytotoxicity of the material (Figure 5I). Next, we studied the anti-tumor effect *in vitro* under various treatment formulations using Live/Dead cell staining (Figure 5A-H). Compared to control groups, 4T1 cells treated with SMID nanoparticles followed by NIR irradiation effectively destructed the majority of tumor cells within the laser irradiation spot, showing a remarkable combination photo-/chemotherapeutic effect (Figure 5G). Addition of H_2_O_2_ as an adjuvant therapeutic further enhanced the tumor ablation effect, owing to the local production of O_2_ and subsequently higher photodynamic yield of cytotoxic ^1^O_2_ (Figure 5H). MTT assays revealed similar findings quantitatively, showing the impressive anti-tumor effect under the photo-induced treatment using SMID nanoparticles supplemented with H_2_O_2_ (Figure 5J). Additionally, reduction of cell viability was positively correlated to the applied drug dosage, with 95.2% of tumor cell death achieved at an equivalent ICG concentration of 16 µg·mL^-1^.

### *In vivo* biodistribution

**Figure 6:**
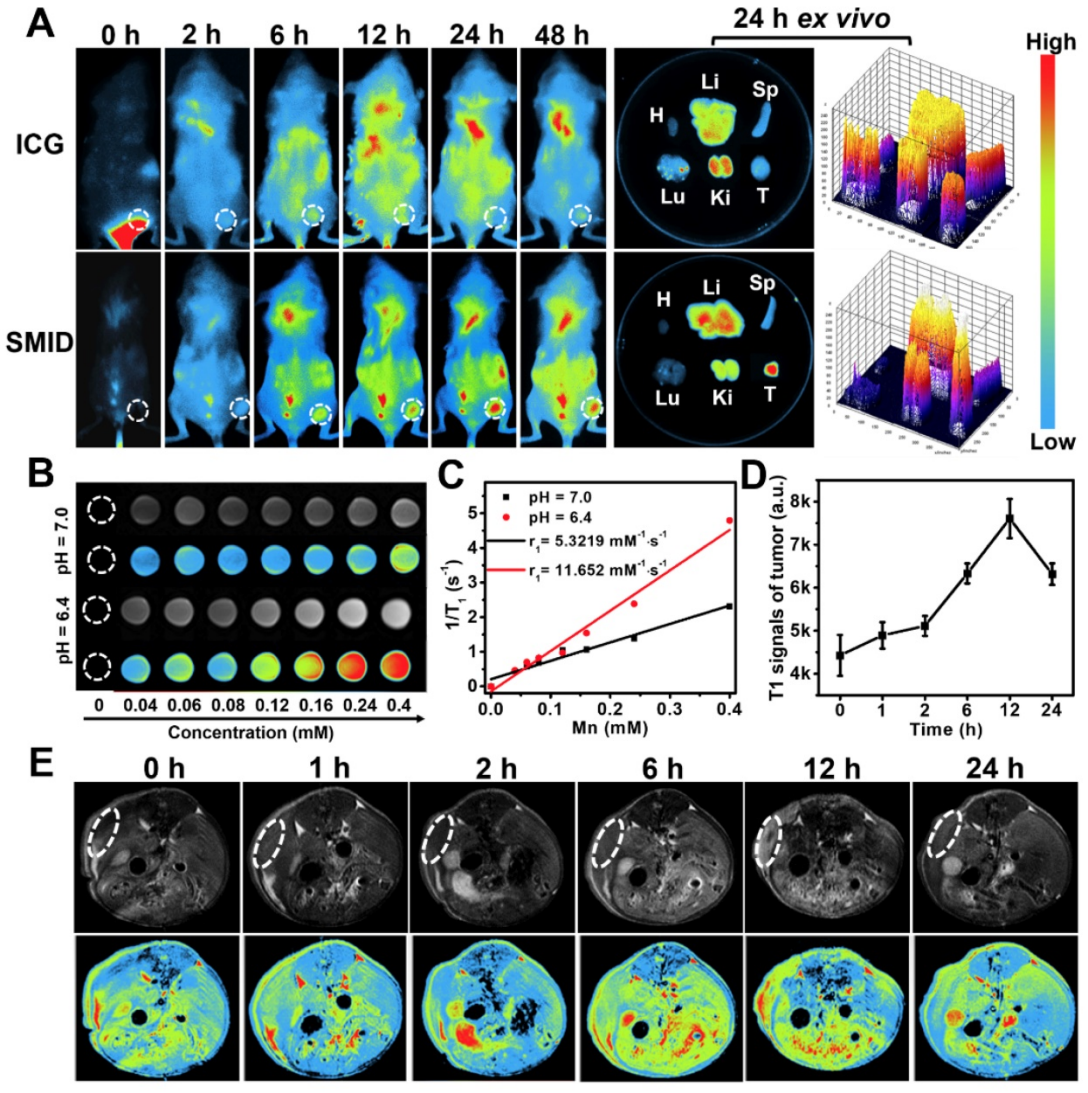
(A) Biodistribution of SMID nanoparticles *in vivo*: fluorescence images of 4T1 tumor-bearing mice taken at different time points after intravenous injection of ICG or SMID nanoparticles (equivalent ICG concentration: 3 mg·kg^-1^), and *ex vivo* fluorescence images and corresponding optical intensity of tumor and major organs (T, Li, Sp, Ki, H, and Lu denote tumor, liver, spleen, kidney, heart, and lung, respectively) dissected at 24 h post-injection; (B) T_1_-weighted greyscale and pseudo-color MR images of SMID nanoparticle dispersions incubated for 12 h under different pH values (6.4 and 7.0); (C) T_1_ relaxation rates as a function of SMID nanoparticle concentration (transverse relativities r1 were 11.652 and 5.322 mM·s^-1^ for SMID nanoparticles at pH 6.4 and 7.0, respectively); (D) quantified MR signals in tumor region after intravenous injection of SMID nanoparticles corresponding to images in (E); (E) T_1_-weighted greyscale and pseudo-color MR images of 4T1 tumor-bearing mice (transverse plane sections) before and after intravenous injection of SMID nanoparticles (equivalent ICG concentration: 3 mg·kg^-1^). White dashed circles denote tumor locations.

Biodistribution of therapeutics *in vivo* is important to evaluate the bioavailability of drugs at target lesion as well as the potential toxicity to normal tissues or organs. We traced the biodistributions of SMID nanoparticles after injection in tumor-bearing mice by NIR fluorescence imaging (Figure 6A). The fluorescence of ICG was widely distributed throughout the mouse body shortly after injection (<12 h). Then, the fluorescence intensity in tumor region gradually intensified (6-24 h) and peaked at 24 h in the SMID-treated group, implying the higher enrichment of nanoparticles in tumor *via* an EPR effect (Figure S23A). Meanwhile, free ICG did not show obvious accumulation at tumors in 48 h after injection. The *ex vivo* biodistribution of drugs was also examined by recording the fluorescence intensity of tumors and major organs dissected from euthanized mice at 24 h post-injection, which further verified the preferential enrichment of ICG at tumor site mediated by SMID nanoparticles. Moreover, strong fluorescence signals from mouse kidneys after injection of free ICG suggested a rapid renal excretion of this small molecule drug. More precisely, the quantitative biodistribution of Mn in mouse body was assessed using inductively coupled plasma atomic emission spectroscopy (ICP-AES) at 2, 8, 12, 24 and 48 h post-injection of SMID nanoparticles (Figure S23B). There was relatively higher accumulation of Mn in reticuloendothelial system, particularly in liver, and in renal system, suggesting the corresponding metabolic pathway for Mn excretion. It was worth noting that the highest Mn level in tumor region was detected as 11.9 % ID/g at 24 h post-injection, which was synchronous with the accumulation of ICG as revealed by fluorescence imaging.

### MR/fluorescence bimodal imaging

It is well-known that Mn atoms in MnO_2_ nanoparticles are coordinated in octahedral geometry to six oxygen atoms and shielded from aqueous environment, while only ionic state of Mn^2+^ can easily access the surrounding water molecules to enhance water proton relaxation and improve MR imaging contrast. MnO_2_ nanoparticles are quite stable in physiological environment, while they decompose into Mn^2+^ and O_2_ under acidic pH condition. As discussed previously, MnO_2_ can also catalyze the decomposition of cancer cell metabolite H_2_O_2_, producing even more Mn^2+^. With five unpaired 3d electrons, Mn^2+^ is a popular T_1_-shorting agent for MR imaging. Therefore, we investigated the MR imaging performance of SMID nanoparticles dispersed in PBS solutions with different pH values (Figure 6B). A notable concentration-dependent brightening effect was found in the T_1_-weighed MR images of SMID nanoparticles under pH 6.4, whereas the MR signals under pH 7.0 appeared to be obviously weaker. Specifically, the r1 value of SMID nanoparticles under pH 6.4 was 11.652 mM·s^-1^, significantly higher than that of 5.322 mM·s^-1^ measured at pH 7.0 (Figure 6C). This r1 value was consistent with those reported in the literature for other Mn^2+^-doped nanoprobes.^34^ Furthermore, MR imaging on 4T1 tumor-bearing mice injected with SMID nanoparticles demonstrated enhanced imaging contrast at tumor site over 12 h (Figure 6D-E), which implied the tumor-specific accumulation of SMID nanoparticles and was generally consistent with the findings by fluorescence imaging *in vivo* (Figure 6A). On the other hand, the peaking of fluorescence intensity (at 24 h) and T_1_-MR signals (at 12 h) at tumor site were asynchronous, which could be due to the faster metabolism of Mn^2+^ than ICG. Nevertheless, these results verified our earlier hypothesis that SMID nanoparticles could be used as a self-traceable agent for fluorescence or MR imaging and detection of tumors, which may effectively aid the imaging-guided evaluation of therapeutic outcome and disease prognosis.

### Anti-tumor effect *in vivo*

**Figure 7:**
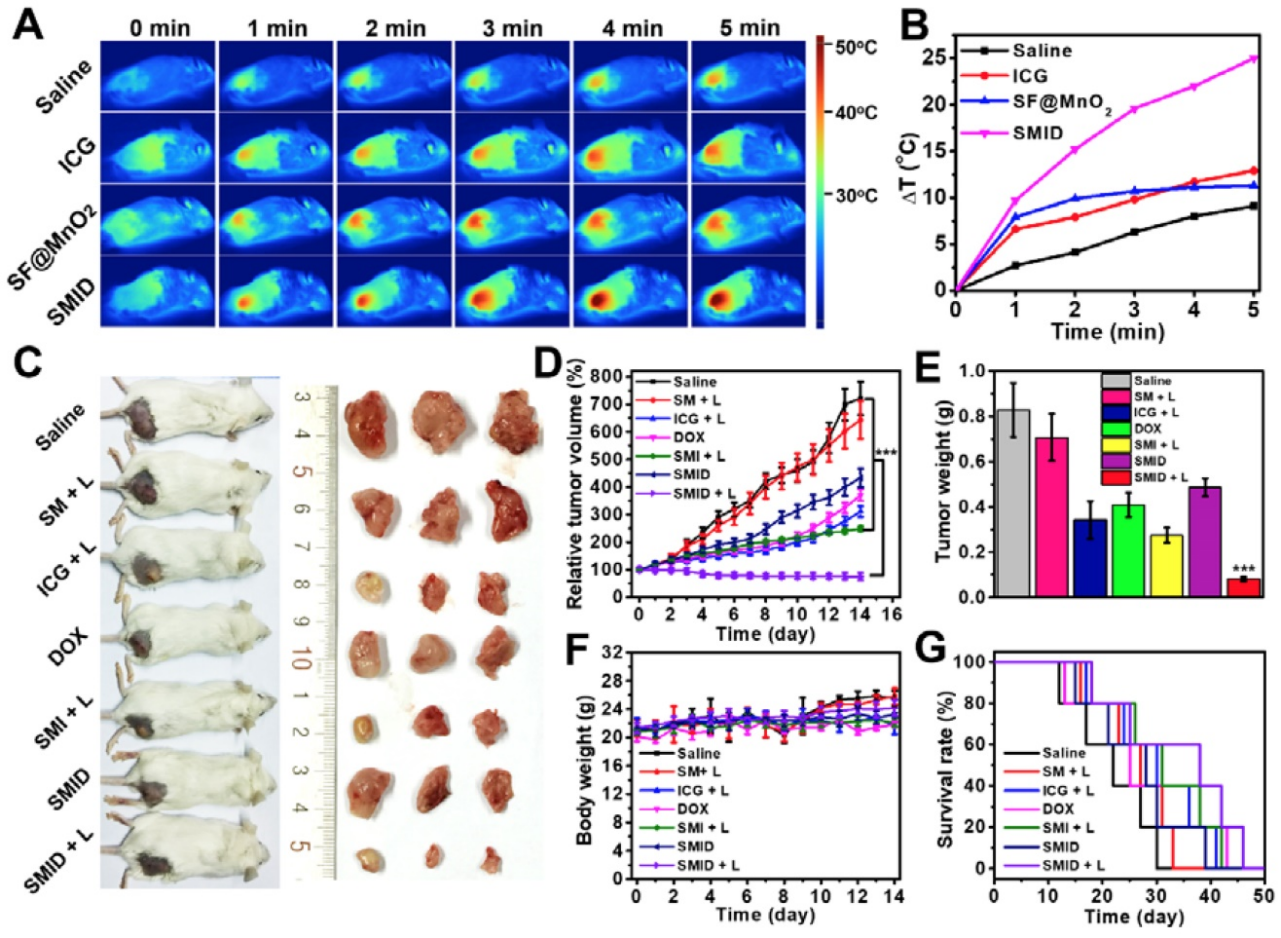
*In vivo* anti-tumor effect in BALB/c mice bearing 4T1 tumors: (A) thermographic images of mouse body during NIR laser irradiation at tumor site for 5 min; (B) change of peak temperature at tumor site over time during NIR irradiation; (C) photographs of treated mice and the corresponding tumors excised at day 14 post-injection; (D) variation of relative tumor volume within 14 days after various treatments (***p<0.001 compared to any other group); (E) average weights of dissected tumors at day 14 (***p<0.001 compared to any other group); (F) variation of mouse body weight within 14 days after various treatments; (G) survival rates of mice subject to various treatments.

The efficacy of SMID nanoparticles for combination PTT/PDT/chemotherapy was evaluated *in vivo* using BALB/c mice bearing 4T1 tumors. At 24 h post-injection, the hyperthermic effect at tumor site was activated by a NIR laser and the temperature of mouse body shell was continuously monitored for 5 min during irradiation (Figure 7A-B). Although the injected SF@MnO_2_ or ICG produced some photothermal effect, the SMID-treated group exhibited the highest temperature elevation at tumor site (25^o^C) in the mice. This remarkable photothermal response *in vivo* was not only attributed to the combination effects of ICG and SF@MnO_2_, but also owing to the higher bioavailability of drug at tumor site as evidenced by MR/fluorescence bimodal imaging (Figure 6). Then, the tumor bearing mice were randomly assigned into six groups that were subject to different treatments, after which the tumor volumes in live mice were measured every day using a caliper (Figure 7D). Compared to the saline group with fast growing tumors, single-modal treatment with chemotherapeutics (DOX, SMID) or photodynamic therapeutics (ICG+L, SMI+L) showed a moderate tumor inhibition effect in 14 days. In contrast, SMID-mediated tri-modal combination therapy (SMID+L) achieved the most significant tumor suppression with a TGI of 89.6%, which was further verified by the smallest anatomical size and weight of the excised tumors among all groups at day 14 (Figure 7C and 7e). Additionally, there was no dramatic weight loss of mice in all treatment groups during 2 weeks of study, suggesting the low systemic toxicity or minimal side effects of the drug formulations (Figure 7F). The median life span of the mice subject to SMID-mediated tri-modal therapy reached 39 days, which was much longer than those in other treatment groups (Figure 7G).

### Histological analysis

**Figure 8:**
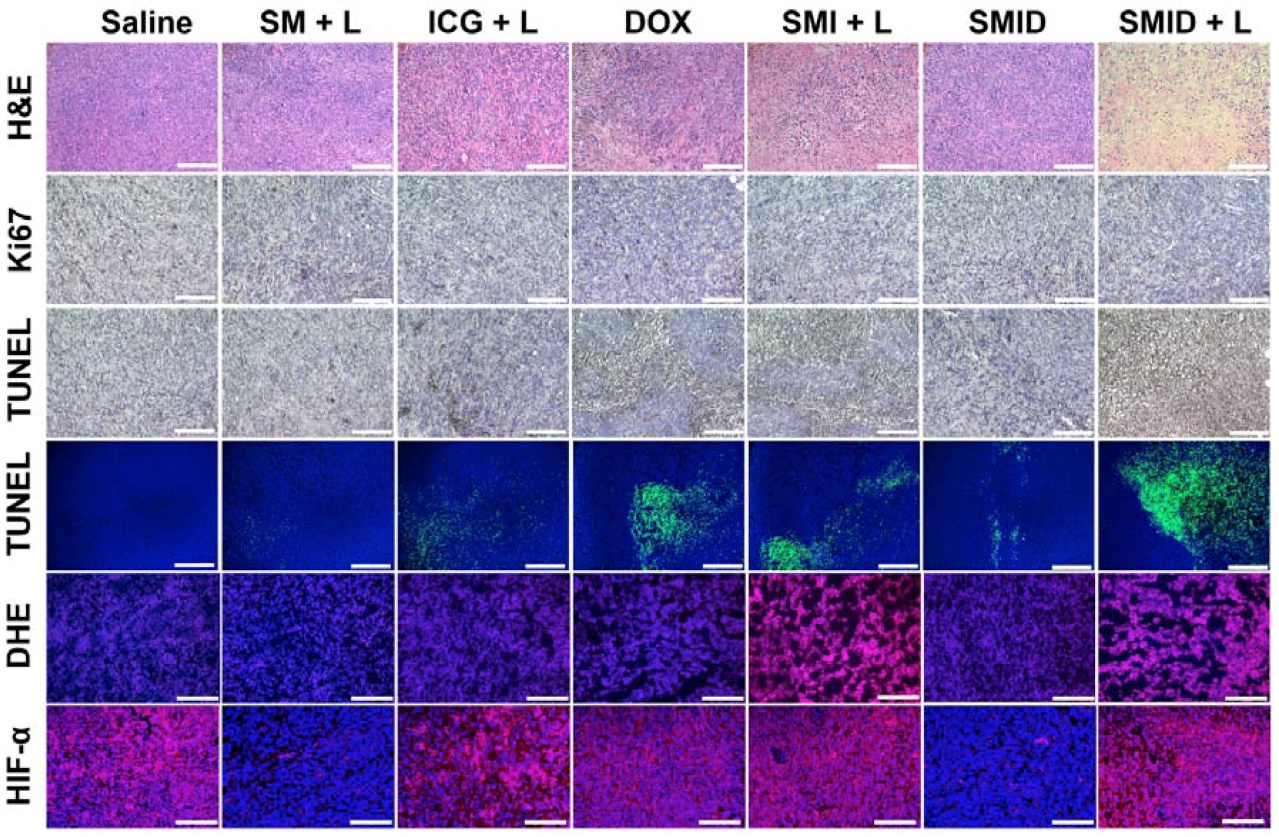
Histopathological analyses of excised tumor sections, including H&E staining, Ki67 staining, colorimetric TUNEL assays (bright field), fluorometric TUNEL assays (fluorescence field), DHE immunofluorescence staining and HIF-α immunofluorescence staining (scale bars: 100 µm).

H&E staining, TUNEL assays, Ki67, DHE and HIF-α immunofluorescence staining of the excised tumor sections were subsequently performed to evaluate the tumor tissue destruction (Figure 8). The most severe tumor cell damages with predominant pyknosis, karyorrhexis and karyolysis was observed in the H&E stained slices of “SMID+L” group, whereas the tumor cells in other treatment groups generally retained their regular morphology with integrative membranes and nuclei. Similarly, the level of proliferating Ki67-positive tumor cells significantly decreased while the apoptotic TUNEL-positive tumor cells dramatically increased in the group of “SMID+L”, indicating a strong anti-tumor effect by induction of cell apoptosis and necrosis. Local ROS generation was verified by DHE staining, which can be oxidized into ethidium under oxidative stress. Then, ethidium can enter the cell nucleus and irreversibly bind with nucleic acids, exhibiting vivid red fluorescence. DHE immunofluorescence staining revealed a distinctly high level of local ROS produced by the ICG-mediated photodynamic process in the groups of “SMI+L” and “SMID+L”. To further confirm the capability of SMID nanoparticles to ameliorate hypoxia inside tumoral tissues, a hypoxyprobe (HIF-α) immunofluorescence assay was performed on tumor slices. Compared to other groups, the Nile red fluorescence was almost absent from the tumor slices in “SM+L” and “SMID” groups, indicating the improved local oxygenation level owing to MnO_2_-catalyzed conversion of endogenous H_2_O_2_ into oxygen. On the other hand, the produced oxygen was consumed during photodynamic therapy in laser-irradiated groups that contains ICG (SMI+L, SMID+L). Such critical change of oxygenation level in tumor microenvironment is particularly beneficial in decreasing the hypoxia‐associated photodynamic resistance during PDT. These histopathological studies verified the strong therapeutic efficacy of SMID-mediated combination PTT/PDT/chemotherapy against 4T1 solid tumors* in vivo*.

### Biosafety evaluation

To study the biosafety of NIR laser irradiation, mice bearing 4T1 tumors were randomly divided into two groups: (1) blank (without any treatment); (2) laser (subject to NIR laser irradiation on tumor region for 5 min). As shown in Figure S24A and S24F, no specific damage such as inflammation, necrosis or ulceration was observed in either tumor region or surrounding normal tissues after laser exposure. The histological sections of major organs did not show any abnormality and there was no irregular change in mouse body weight (Figure S24E-F), indicating acceptable biosafety of the applied NIR laser power (2 W·cm^-2^) for antitumor applications. Moreover, there was no significant difference in tumor growth rate between these two groups during 14 days post-administration, suggesting that exposure to NIR laser alone induced no obvious tumor suppression effect (Figure S24B-D). The potential toxicity of SMID nanoparticles was assessed in terms of hemocompatibility and histocompatibility. The percentage of hemolytic erythrocytes were generally less than 5% after 6 h of incubation with SMID nanoparticles under the concentration from 50 to 400 µg·mL^-1^ (Figure S25). Furthermore, the complete counts of key blood components and other principle hematology parameters of the treated mice were all within the reference ranges of healthy mice in two weeks after injection (Figure S26). For histological analysis, the major organs of treated mice were excised at day 14 and processed by H&E staining. The stained sections from the mice subject to different treatment formulations showed no obvious inflammation or lesions at cellular or tissue levels (Figure S27). These characterizations suggested that SMID nanoparticles posed minimal systemic toxicity or adverse effect for *in vivo* applications.

## Conclusions

In summary, MnO_2_ was mineralized onto silk fibroin (SF) nanoparticles through a bioinspired crystallization process in an extremely facile way, taking advantage of the unique properties of SF as both a template and a reductant. The present study also demonstrated the strong and stable photothermal response of SF@MnO_2_ composite nanocarrier, which was loaded with ICG/DOX as a multifunctional theranostic platform (SMID) and realized potent combination PTT/PDT/chemotherapy under the guidance of fluorescence/MR imaging. This nanotherapeutic exhibited enhanced bioavailability at tumor site after intravenous administration and was responsive to various tumor-specific endogenous and exogenous stimuli. Particularly, MnO_2_-catalyzed decomposition of endogenous H_2_O_2_ into O_2_ relieved the hypoxia in tumor microenvironment and thus significantly enhanced the efficacy of PDT. Meanwhile, the produced Mn^2+^ considerably improved the T_1_-MR contrast of tumoral tissues, allowing the MRI-guided theranostics. Animal studies based on tumor-bearing mice confirmed the remarkable anti-tumor effect and biocompatibility of SMID nanoparticles *in vivo*. This SF@MnO_2_-based nanocarrier demonstrated a promising potential for integrating multi-modal theranostic agents for cancer therapy. For instance, targeting ligands such as antibodies, peptides, and small molecules (e.g. folate and iRGD) could be conjugated onto SF@MnO_2_ nanocarrier for targeted drug delivery in clinical applications. Of course, as many other nanotherapeutics, systematic pre-clinical and clinical investigations are necessary to fully understand the biosafety of SMID nanoparticles in large animals and human body. In addition, this strategy may also be generally extended to dope other metallic compounds on SF for various applications in the fields of biomedicine, catalysis and energy.

## Methods

### Materials

*Bombyx mori* cocoons were provided by the Institute of Sericulture and Systems Biology, Southwest University (China). Potassium Permanganate (KMnO4) was obtained from Chongqing Chuandong Chemical (group) Co., LTD (China). Fluorescein diacetate (FDA), formalin solution (neutral buffered, 10%), 1, 3-diphenyl-isobenzofuran (DPBF) were obtained from Sigma-Aldrich (USA). Doxorubicin (DOX) was purchased from Beijing Hvsf United Chemical materials Co., LTD (China). Hydrogen peroxide (H_2_O_2_, 30%), calcium chloride dehydrate (CaCl_2_·2H_2_O, >99%), hydrochloric acid (HCl, 37%), sodium hydroxide (NaOH, >97.0%), indocyanine green (ICG), thiazolyl blue tetrazolium bromide (MTT, 98%), glutathione (GSH), trichloroacetic acid (TCA), chlorpromazine (CPZ), nystatin, amiloride, methyl-beta-cyclodextrin (Me-β-CD) and dimethyl sulfoxide (DMSO) were obtained from Shanghai Aladdin Bio-Chem Technology Co., Ltd. (China). H_2_O_2_ quantitative assay kit was purchased from Sangon Biotech (Shanghai) Co., LTD (China). Dulbecco's modified eagle's medium (DMEM), TrypLETM Express enzyme, 4',6-Diamidino-2-Phenylindole (DAPI), fetal bovine serum (FBS), penicillin (10000 unit·mL^-1^)/streptomycin (10000 μg·mL^-1^), LIVE/DEAD^®^ viability/cytotoxicity kit, LysoTracker Red DND-99, singlet oxygen sensor green (SOSG), 5,5'-dithiobis (2-nitrobenzoic acid) (DTNB) and phosphate buffered saline (PBS) were acquired from Thermo Fisher Scientific, Inc. (USA). 2', 7'-Dichlorofluorescein diacetate (DCFH-DA) was obtained from GEN-VIEW Scientific Inc. (USA). Rabbit Anti-HIF-1 Alpha antibody was purchased from Beijing Biosynthesis Biotechnology, LTD (China). TUNEL Apoptosis Assay Kit, Ki67 Cell Proliferation Kit and Proteinase K were purchased from Beyotime Biotechnology (China). KM mice and BALB/c mice models were purchased from Chongqing Teng Xin Bill Experimental Animal Sales Co. Ltd (China). Deionized (DI) water (18.2 MΩ·cm) was collected from a Milli-Q Synthesis A10 purification system (Molsheim, France).

### Preparation of SF solution from *Bombyx mori* cocoons

SF solution was prepared according to a standard method as previously reported with further optimization 65, 66. Briefly, *Bombyx mori* cocoons were stripped from the silkworm chrysalis and were subsequently sliced into thin pieces with 1~2 cm^2^ in size. Then, the cocoon slices were incubated with 0.5% Na_2_CO_3_ solution under the boiling state for 30 min to remove the gum-like silk sericin protein. The flocculent precipitation were thoroughly rinsed with DI water and dried in a drying cabinet at 35^o^C overnight. After degumming, the silk fibers were immersed in Ajisawa's reagent containing CaCl_2_, ethanol, and DI water (molar ratio at 1:2:8) for 2 h at 90^o^C. The impurities including small molecules and salts were eliminated from the silk solution by standard dialysis using Slide-a-Lyzer dialysis cassette (MWCO 3500, Pierce) for 3 days. The obtained regenerated SF solution was stored at 4 °C for further use.

### Synthesis of SMID nanoparticles

The preparation of SF nanoparticles was based on a previously reported method with slight modifications 67, 68. Briefly, regenerated SF solution (2.0 wt%) was rapidly introduced into acetone (1:9, v/v) under vigorous stirring at room temperature to obtain water-miscible acetone. The milk-like mixture was stirred for at least 8 h at room temperature to remove acetone, and the resulting SF nanoparticles were harvested after a desolvation process. The products were stored at 4^o^C and ready for use.

For bioinspired crystallization of MnO_2_ on the surface of SF nanoparticles, 1 mL of KMnO_4_ solution (5 mg·mL^-1^) was dropwise added into 20 mL of SF nanoparticle dispersion (500 μg·mL^-1^). Then, the mixed solution was stirred at room temperature for 15 min, followed by removing the unreacted KMnO_4_ by centrifugation. The precipitates were re-suspended in DI water, and SF@MnO_2_ nanoparticles were finally obtained after washing twice. For drug loading, ICG (6 mg) and DOX (6 mg) were firstly dissolved in 10 mL of DI water, which was stirred for 30 min under absolutely dark condition 68. Afterwards, 20 mL of SF@MnO_2_ suspension (400 μg·mL^-1^) was introduced into the as-prepared drug mixture under stirring for 6 h. After high-speed centrifugation at 8,000 rpm for 5 min and washing for three times, SMID nanoparticles were obtained and re-dispersed in DI water before use.

### Characterizations of SMID nanoparticles

The morphology of SMID nanoparticles and intermediate products of each synthetic step was recorded by both transmission electron microscopy (TEM, JEM-2100, JEOL, Japan) and field emission scanning electron microscopy (FESEM, JSM-7800F, JEOL, Japan). The hydrodynamic diameter and surface potential of various products were measured by dynamic light scattering (DLS) using Zetasizer (Nano ZS90, Malvern Instruments, UK). The UV-vis-NIR absorbance spectra were analyzed using a spectrometer (UV-1800, Shimadzu, Japan) and the fluorescence emission spectra were detected by a fluorescence spectrophotometer (RF-530l, Shimadzu, Japan). The Fourier transform infrared (FT-IR) spectra were measured using a FT-IR spectrophotometer (Nicolet 6700, Thermo Scientific, USA). The crystalline form and purity of the products were characterized using an X-ray diffractometer (XRD-7000, Shimadzu, Japan) with CuK_α_ radiation (λ=1.5406 Å). The Raman spectra were recorded using LabRAM HR Evolution (HORIBA, Japan). The surface chemistry of SF nanoparticles before and after bioinspired mineralization was determined by X-ray photoelectron spectroscopy (XPS) using an X-ray photoelectron spectrometer (ESCALAB 250Xi, Thermo Fisher Scientific, USA). The amount of mineralized MnO_2_ was quantified based on thermogravimetric analysis (TGA) using a thermogravimetric analyzer (WRNK-529, Shanghai Automation Instrumentation, Co. Ltd, China). Nitrogen adsorption-desorption isotherms were measured to determine the Brunauer-Emmett-Teller (BET) surface areas and pore volumes using a Quantachrome Nova 1200e analyzer.

### Drug loading efficiency and *in vitro* drug release

To determine the drug loading content (DLC) and encapsulation efficiency (EE), the optical spectra of supernatants collected after each washing step were measured using a Shimadzu UV-1800 spectrophotometer. The amount of ICG and DOX in the supernatants were determined according to the corresponding calibration curves (absorbance intensity vs. concentration) at 488 nm and 780 nm, respectively. Then, the DLC and EE for both drugs were calculated according to formulas (4) and (5), respectively.


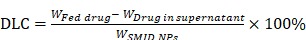
 (4)


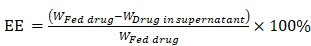
 (5)

where W_Fed drug_ is the initial amount of fed drug, W_Drug in supernatant_ is the amount of drug in the supernatants after centrifugation, and W_SMID NPs_ is the amount of as-synthesized SMID nanoparticles.

Drug release from SMID nanoparticles was evaluated under different pH or H_2_O_2_ conditions based on a traditional dialysis method *in vitro* 69. Specifically, 2 mL of SMID nanoparticle dispersion (1.5 mg·mL^-1^) was loaded into each dialysis bag (MWCO = 3500) and then submerged into 78 mL of 1×PBS as releasing medium under different pH conditions (pH = 7.4, 6.8 or 5.0). H_2_O_2_ concentration was set as 100 μM to reflect the condition of tumor microenvironment in the H_2_O_2_ positive groups. The setup was placed in a shaker at a constant temperature of 37 °C or 43 °C under the dark condition. To monitor the release kinetics, 3 mL of releasing medium was sampled at predesigned time points, and fresh medium of equivalent volume was supplemented to the releasing system. To analyze the photothermal response of drug release, the dialysis bag was exposed to an NIR laser (808 nm, 2 W·cm^-2^) for several on/off cycles (5 min of irradiation per cycle). The amount of released drug was quantified according to a calibration curve measured by florescence spectroscopy (λ_ex_: 488 nm, λ_em_: 560 nm).

### Reactivity of SF@MnO_2_ nanoparticles to H_2_O_2_ *in vitro*

O_2_ generation from H_2_O_2_ catalyzed by SF@MnO_2_ nanoparticles was measured in a sealed chamber integrated with an oxygen probe using a portable digital LCD dissolved oxygen meter (JPB-607A, Shanghai INESA Scientific Instrument, Co. Ltd, China). Specifically, H_2_O_2_ (30 wt%) was diluted by deoxygenated water to 1 mM, and 2 mg of dried SF@MnO_2_ nanoparticles were redispersed in 1 mL of deoxygenated water. Then, SF@MnO_2_ nanoparticle dispersion were rapidly added into 20 mL of H_2_O_2_ diluent under stirring at room temperature. The dissolved O_2_ in H_2_O_2_ solution was recorded at the interval of 10 sec. To monitor the pH variation, a pH electrode was incorporated during the measurement using a pH meter (PHSJ-5, Shanghai INESA Scientific Instrument, Co. Ltd, China) while maintaining the same testing conditions as in the dissolved oxygen assay. To investigate the consumption of H_2_O_2_ solution, 1 mL of SF@MnO_2_ nanoparticle dispersion at various concentrations were incubated with 20 mL of H_2_O_2_ solution (1 mM) for 30 min. The percentage of unreacted H_2_O_2_ was quantified using a H_2_O_2_ quantitative assay kit (water-compatible) following the manufacturer's protocol.

### Photothermal property of SMID nanoparticles *in vitro*

To investigate the NIR-light induced temperature elevation, 3 mL of sample solution was loaded in a transparent quartz vial and irradiated by a fiber-coupled semiconductor diode NIR laser (808 nm) at various output power density for 10 min. The temperature probe of a digital thermometer was immersed into the sample to record the local temperature at the time interval of 10 sec. The photothermal stability of SMID nanoparticles was analyzed through periodical on/off laser irradiation for 5 cycles. Briefly, the sample-laden cuvette was exposed to an NIR laser for 10 min, followed by cooling under room temperature prior to the next irradiation cycle. The NIR-light induced temperature variation of SMID nanoparticle dispersions at gradient concentrations was also imaged by color mapping using a thermal imaging camera (Fluke, TiS55).

### Reactive oxygen species (ROS) yield of SMID nanoparticles

A fluorescence probe, DPBF, was used to detect reactive oxygen species (ROS) produced in photodynamic reaction *in vitro*. Briefly, 45 μL of DPBF (59.5 µM) acetonitrile dispersion was added into 3 mL of aqueous dispersion of ICG, SF@MnO_2_ nanoparticles or SMID nanoparticles (equivalent ICG concentration: 10 μg·mL^-1^) upon rigorous mixing. Then, the mixture was transferred into a cuvette (optical path: 1 cm), which was further irradiated by a NIR laser (808 nm, 2 W·cm^-2^) for 10 min. Thereafter, UV-vis absorption spectra of each sample was measured at different time points during laser exposure. The amount of ROS generation was positively correlated to the decreasing rate of optical absorbance intensity at 417 nm. Moreover, the ROS generation under laser irradiation with or without H_2_O_2_ solution (100 µM) was measured using an SOSG probe. Briefly, 3 µL of SOSG methanol solution (5 mM) was introduced into 3 mL of aqueous dispersion, which was further irradiated by an NIR laser for 10 min. After that, SOSG fluorescence (λ_ex_: 494 nm, λ_em_: 532 nm) was measured to determine the generated ^1^O_2_.

Intracellular ROS generation was detected using DCFH-DA or SOSG probe following a standard protocol. Briefly, 4T1 tumor cells were seeded in a 12-well plate (1×10^5^ cells per well) and incubated for 12 h. Then, the cells were incubated with various agents for 4 h, followed by NIR laser irradiation (808 nm, 2 W·cm^-2^) for 10 min with or without H_2_O_2_ solution (100 µM). Next, the cells were stained with DCFH-DA (5 μg·mL^-1^) or SOSG (5 μg·mL^-1^) for 30 min and DAPI (1 µg·mL^-1^) for 10 min. After rinsing with 1×PBS for three times, fluorescence images of the cells were acquired by confocal laser scanning microscopy (CLSM, LSM800, Zeiss, Germany). DCFH-DA probe was also utilized to monitor the intracellular H_2_O_2_ consumption. Briefly, 4T1 cells were seeded in a 12-well plate (1×10^5^ cells per well) and incubated for 12 h. Then, the culture medium was replaced by DMEM containing 1mM H_2_O_2_, followed by another incubation for 4 h. Afterwards, cells were further treated by various agents (equivalent ICG concentration: 10 μg·mL^-1^) for 4 h, and were stained by DCFH-DA and DAPI for CLSM.

### GSH consumption efficiency *in vitro*

GSH consumption efficiency was analyzed using standard Ellman reagent. Briefly, 40 mg DTNB were dissolved in 10 mL PBS (0.1 M) buffer solution containing 1 mM EDTA (pH = 8.0). 1 mL of SF@MnO_2_ nanoparticles at gradient concentration was allowed to react with 1 mL of GSH dispersion (0.2 mg·mL^-1^) at room temperature for 4 h. Then, 400 µL of the as-prepared DTNB solution was introduced into the above system. After incubation for 30 min, all the samples were added into a 96-well plate and the optical absorbance at 412 nm was measured using a microplate reader (Infinite M200 PRO, TECAN). To measure the SF@MnO_2_ activated intracellular GSH consumption, 4T1 tumor cells were firstly seeded in a 96-well plate (1×10^4^ cells per well) and incubated for 12 h. Then, the cells were incubated with gradient concentrations of SF@MnO_2_ nanoparticles for 4 h. Then, the cells were treated by 50 µL of 6.5% TCA solution at 4^o^C for 30 min. Finally, 250 µL of the Ellman reagent was introduced into each well. After the reaction under gent shaking for 10 min, optical absorbance of each well was similarly measured at 412 nm.

### Biocompatibility evaluation of SMID nanoparticles *in vitro*

To assess the biocompatibility of SF@MnO_2_ nanocarriers, typical MTT cell viability assay was conducted on both humanized umbilical vein endothelial cells (HUVECs) and murine L929 fibrosarcoma cells (L929 cells) *in vitro*. Specifically, HUVECs and L929 cells at an initial seeding density of 1×10^4^ per well were cultured in a 96-well cell culture plate at 37^o^C overnight. Then, the cells were incubated with 200 μL of SF@MnO_2_ nanoparticles with gradient concentrations (25~1600 μg·mL^-1^) for 24 h. Afterwards, the supernatant was discarded, followed by adding 200 μL of MTT solution (250 μg·mL^-1^) into each well. After 6 h of incubation, 150 μL of DMSO was introduced to replace the previous supernatant, followed by gently shaking for 15 min. Finally, the optical absorbance intensity at 490 nm and 630 nm was measured using a microplate reader (SPARK 10M). The cell viability was calculated according to formula (6):


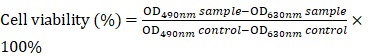
 (6)

The hemolytic potential of SMID nanoparticles was assessed using the peripheral blood sampled from orbital venous plexus of KM mice. The whole blood was centrifuged at 3000 rpm for 5 min, and the precipitate of erythrocytes was rinsed with 1×PBS four times. Then, 0.5 mL of erythrocytes (4% v/v) was mixed with 0.5 mL of SMID nanoparticles dispersed in 1×PBS at gradient concentrations (50, 100, 200 and 400 µg·mL^-1^), followed by incubation at 37 °C for 6 h. Finally, all the specimens were centrifuged at 10,000 rpm for 10 min, and the absorbance intensity of supernatant at 570 nm was recorded using a UV-vis spectrophotometer (Shimadzu UV-1800, Japan).

### Cellular uptake and internalization

Cellular uptake of SMID nanoparticles was investigated using flow cytometry. Briefly, 4T1 tumor cells with an initial seeding density of 7.5×10^4^ cells per well were incubated in a 24-well plate at 37 °C for 12 h. Then, fresh culture medium containing SMID nanoparticles (equivalent ICG concentration: 10 μg·mL^-1^) was introduced to replace the old medium, followed by further incubation for 0.5 h, 1 h, 2 h and 4 h. The untreated cells were cultured in parallel as blank control. Afterwards, the 4T1 cells were digested with TrypLE Express enzyme and resuspended in 400 µL of 1×PBS containing Ca^2+^ and Mg^2+^, and were analyzed using a flow cytometer (NovoCyte TM 2060R, ACEA Biosciences, USA). To explore the underlying mechanism of cellular uptake of SMID nanoparticles, 4T1 cells were pretreated by NaN_3_ (0.1%, w/v), Nystatin (50 μg·mL^-1^), amiloride (2 mM), CPZ (20 μg·mL^-1^) and Me-β-CD (0.5 mM), followed by incubation with SMID nanoparticles (equivalent ICG concentration: 10 μg·mL^-1^) at 37^o^C for 4 h. The cellular uptake efficiency under various conditions was analyzed similarly using flow cytometry.

For fluorescence imaging, 4T1 cells were treated with DOX, ICG and SMID nanoparticles (equivalent ICG concentration: 10 μg·mL^-1^) for various periods of time. Then, the cells were rinsed with 1×PBS for three times and stained with DAPI (1 µg·mL^-1^) for 5 min. After that, fluorescence images of the treated cells were captured using a fluorescence microscope (IX73, Olympus, Japan). To investigate the lysosomal co-localization and escape, 4T1 cells were seeded on a 35-mm Petri dish at a density of 3×10^4^ cells and incubated at 37^o^C. Upon reaching a culture confluency of 50%-70%, cells were further incubated with SMID nanoparticles (equivalent ICG concentration: 10 μg·mL^-1^) for various periods of time. Then, the cells were stained with DAPI (1 µg·mL^-1^) for 5 min and Lyso Tracker Red DND-99 (50 nM) for 45 min. Finally, fluorescence images of stained cells were captured using CLSM.

### *In vitro* cytotoxicity

Photo-induced cytotoxicity of SMID nanoparticles was evaluated by fluorescence staining. Briefly, cells with initial seeding density of 1×10^5^ cells per well were incubated in a 12-well plate for 12 h. Then, the cells were incubated with 800 µL of DOX, ICG and SMID nanoparticles (equivalent ICG concentration: 16 μg·mL^-1^) for another 4 h, followed by exposure to an NIR laser (808 nm, 2 W·cm^-2^) for 5 min. After incubation for another hour, the cells were stained with LIVE/DEAD^®^ viability/cytotoxicity kit following the manufacturer's protocol. The fluorescence images of stained cells were recorded under a fluorescence microscope (IX73, Olympus, Japan). To evaluate the therapeutic effect of SMID nanoparticles against tumor cells *in vitro*, 4T1 cells with an initial seeding density of 1×10^4^ per well were cultured in a 96-well plate for 12 h. Then, the cells were incubated with 200 µL of various agents (equivalent ICG concentration: 16 μg·mL^-1^) for 4 h, and were irradiated by an 808 nm laser (2 W·cm^ -2^) where applicable. After culturing for 12 h, cell viability was determined based on standard MTT cell viability assay.

### Establishment of 4T1 tumor bearing BLAB/c mouse model

All animal experiments were performed under the supervision of Institutional Animal Care and Use Committee (IACUC) of Southwest University and in compliance with the National Guide for Care and Use of Laboratory Animals. 4T1 tumors was established in female BALB/c mice 70. Briefly, the mice (6 weeks, ~25 g each) were subcutaneously inoculated with 4T1 cells (1×10^6^ in saline) on dorsal side. After about 10 days, mice with a tumor grown to ~200 mm^3^ were grouped for antitumor treatments. The tumor volume was continuously monitored thereafter and calculated according to formula (7).

Tumor volume = (Tumor length) × (Tumor width)^2^ × 0.5 (7)

where length represents the longest dimension and width denotes the shortest dimension of a tumor.

### Biodistribution *in vivo*

Biodistribution of SMID nanoparticles was investigated by quantifying the content of Mn element in mouse body. Briefly, 4T1 tumor-bearing BALB/c mice were intravenously injected with 200 µL of SMID (equivalent ICG dosage: 3 mg·kg^-1^) through the tail vein. Then, the mice were sacrificed to harvest the tumor and major organs at predesignated time points (2, 8, 12, 24 and 48 h) post-injection. The samples were lysed using chloroazotic acid, and the Mn content was analyzed using inductively coupled plasma atomic emission spectroscopy (ICP-AES).

### MR/fluorescence dual-modal imaging *in vivo*

To determine the biodistribution of SMID nanoparticles by fluorescence imaging *in vivo*, tumor bearing BALB/c mice were intravenously injected with free ICG or SMID nanoparticles (equivalent ICG concentration: 3 mg·kg^-1^). At 2, 6, 12, 24 and 48 h after injection, infrared fluorescence images of mice were acquired using a fluorescence imaging system (λ_ex_ = 780 nm, λ_em_ = 830 nm, Fusion FX7 Spectra, VILBER, France). At 24 h post-injection, mice were sacrificed to harvest the major organs and tumors for *ex vivo* imaging to determine the biodistribution of SMID nanoparticles semi-quantitatively using the same luminescence imaging system. MRI was carried out using Siemens MAGNETOM Prisma 3.0 T MRI scanner (Erlangen, Germany) with gradient strength up to 80 mT/m (TR = 800 ms, TE = 12 ms). T_1_-weighted MR signal intensity of each sample was measured from the MR images in the region of interest (ROI). The relaxation rate r_1_ (1/T_1_) was then calculated from T_1_ values under different Mn^2+^ concentrations. For MRI *in vivo*, T_1_-weighted MR images of tumors were taken at 1, 2, 6, 12, 24 h after intravenous injection of SMID nanoparticles (equivalent ICG concentration: 2 mg·kg^-1^).

### Therapeutic efficacy of SMID nanoparticles *in vivo*

The 4T1 tumor bearing mice was randomly divided into 7 groups (n = 5 for each group): (1) saline, (2) SF@MnO_2_ plus laser irradiation, (3) free ICG plus laser irradiation, (4) free DOX, (5) SF@MnO_2_/ICG plus laser irradiation, (6) SMID, (7) SMID plus laser irradiation. The equivalent dose of ICG for all groups was fixed at 3 mg·kg^-1^. At 24 h post-injection, NIR laser irradiation (808 nm, 2 W·cm^-2^) was conducted at the tumor region for group (2), (3), (5) and (7) for 5 min. To monitor the temperature change, an infrared thermal camera (TiS55, Fluke, USA) was used to capture the thermal images of mice at predetermined time points. After the treatments, tumor volume and mouse body weight were continuously monitored for 14 days. The tumor growth inhibition (TGI) rate was indexed according to formula (8).

TGI = (V_C_-V_T_)/V_c_ × 100% (8)

where V_C_ stands for the tumor volume of the saline group, and V_T_ denotes the tumor volume in treatment groups.

### Histological analysis

At day 14 after various treatments, the mice were euthanized to excise tumor and major organs, including hearts, livers, spleens, lungs and kidneys. The tumors were weighed and all the excised tissues were sliced into sections (5 µm) for histological analysis. The histological sections of tumor were formalin-fixed and paraffin-embedded, followed by staining with hematoxylin & eosin (H&E), Ki67, terminal deoxynucleotidyl transferase dUTP nick end labeling (TUNEL) and HIF1-α following the manufacturer's protocol. In addition, the tumor sections were alternatively embedded in OCT medium and snap frozen, followed by staining with dihydroethidine (DHE). All stained sections were histologically examined under a fluorescence microscope (IX73, Olympus, Japan).

### Statistical analysis

Data were presented as mean ± SD according to one-way analysis of variance (ANOVA) using OriginPro 8.5 software (OriginLab, MA, USA). A *p*-value less than 0.05 (^*^p<0.05, n = 4) was considered as statistically significant.

## Supplementary Material

Supplementary figures and methods.

## Abbreviations

DLS: dynamic light scattering
DOX: doxorubicin
DLC: drug loading content
DLS: dynamic light scattering
EE: encapsulation efficiency
EPR: enhanced permeability and retention
FBS: fetal bovine serum
ICG: indocyanine green
GSH: glutathione
H&E: hematoxylin and eosin
MR: magnetic resonance
NDDS: nanoscale drug delivery systems
PBS: phosphate buffer saline
PDT: photodynamic therapy
PTT: photothermal therapy
SF: silk fibroin
SEM: scanning electronic microscopy
SOSG: singlet oxygen sensor green
TUNEL: terminal deoxynucleotidyl transferase dUTP nick end labeling
XRD: X-ray powder diffraction
XPS: X-ray photoelectron spectroscopy
