# Supplementary material for: Biomineralization-inspired Crystallization of Manganese Oxide on Silk Fibroin Nanoparticles for in vivo MR/fluorescence Imaging-assisted Tri-modal Therapy of Cancer
Source: Theranostics. 2019 Aug 14;9(21):6314–33. doi: 10.7150/thno.36252 (PMC6735506; doi:10.7150/thno.36252)
Supplement: Supplementary file 1 — Supplementary figures and methods. [file thnov09p6314s1.pdf]

Supplementary Material

Biomineralization-inspired Crystallization of Manganese Oxide on Silk Fibroin Nanoparticles for *in vivo* MR/fluorescence Imaging-assisted Tri-modal Therapy of Cancer

Ruihao Yang [1, 2], Mengmeng Hou [1, 2], Ya Gao [1, 2], Shiyu Lu [1], Lei Zhang [3], Zhigang Xu [1, 2], Chang Ming Li [1], Yuejun Kang [1, 2, *], Peng Xue [1, 2, *]

[1] Key Laboratory of Luminescent and Real-Time Analytical Chemistry (Southwest University), Ministry of Education, School of Materials and Energy, Southwest University, Chongqing 400715, China.

[2] Chongqing Engineering Research Center for Micro-Nano Biomedical Materials and Devices, Southwest University, Chongqing 400715, China.

[3] Institute of Sericulture and Systems Biology, Southwest University, Chongqing 400716, China.

Corresponding authors

* E-mail: xuepeng@swu.edu.cn (P. Xue)

* E-mail: yjkang@swu.edu.cn (Y. Kang)

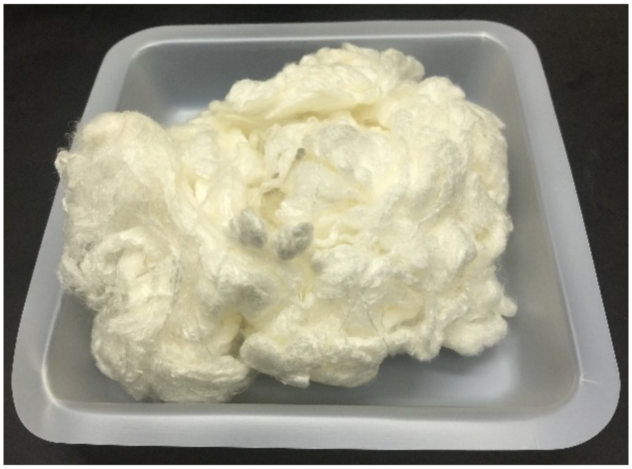

Figure S1. Regenerated silk fibroin (SF) obtained after a standard degumming process.

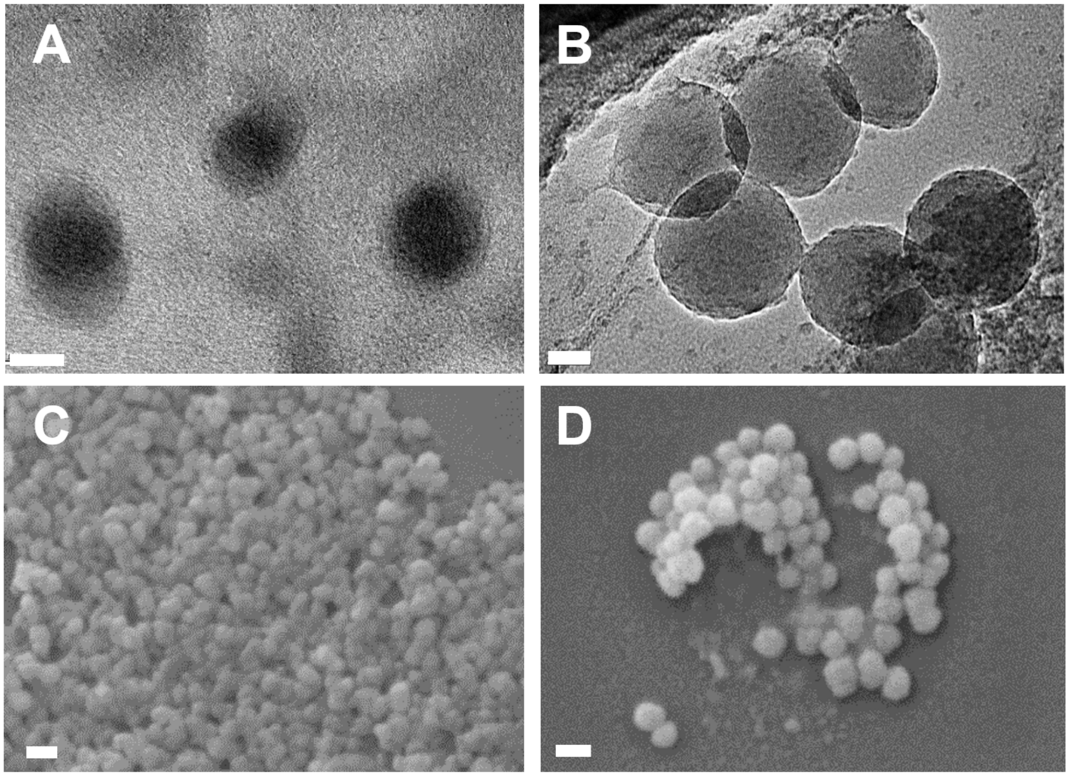

Figure S2. TEM images of (A) SF and (B) SF@MnO_2 nanoparticles (scale bars: 20 nm); SEM images of (C) SF and (D) SF@MnO_2 nanoparticles (scale bars: 100 nm).

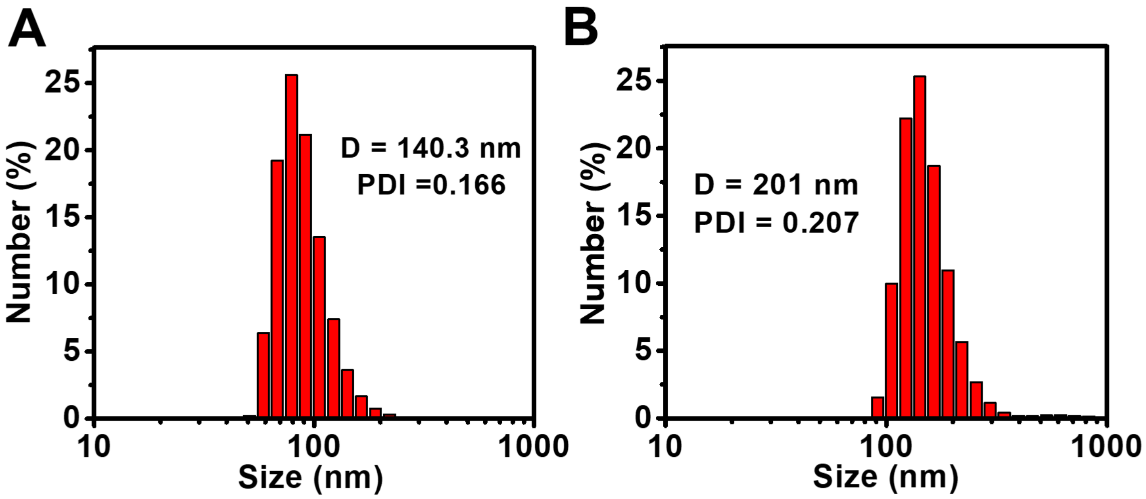

Figure S3. Hydrodynamic size distributions of (A) SF and (B) SF@MnO_2 nanoparticles measured by DLS.

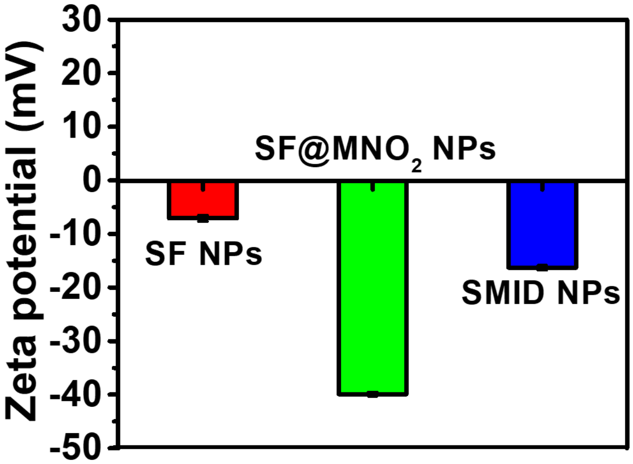

Figure S4. Zeta potentials of SF, SF@MnO_2 and SMID nanoparticles.

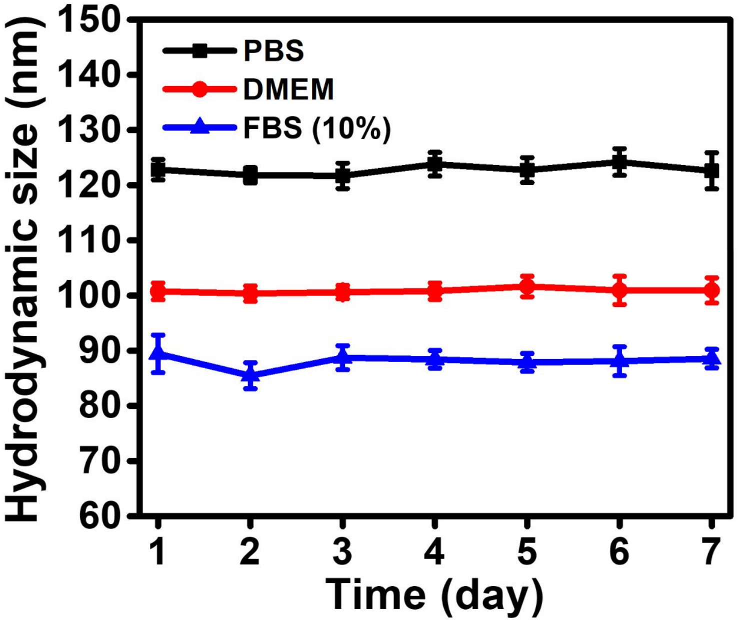

Figure S5. Size variation of SMID nanoparticles dispersed in PBS, DMEM or FBS (10%) during 7 days.

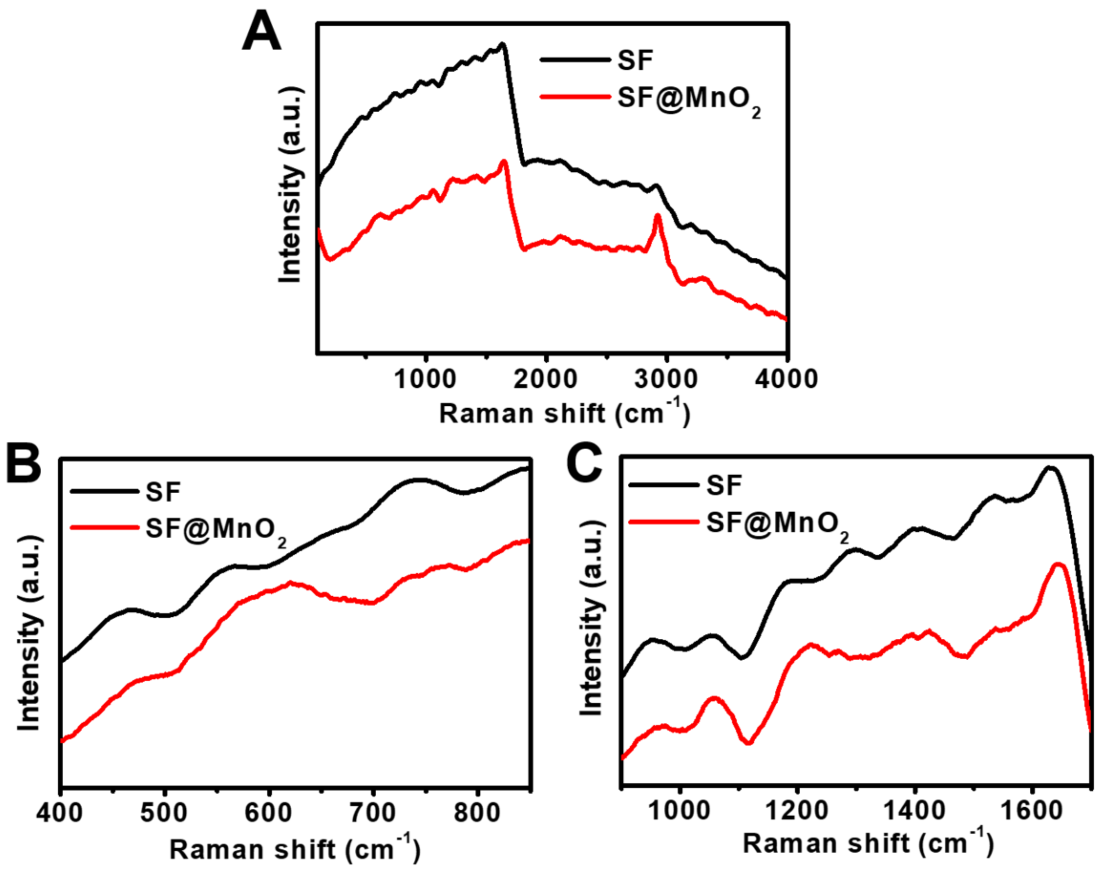

Figure S6. Raman spectra of SF and $SF@MnO_2$ nanoparticles: (A) a full spectrum in 100 ~ 4000 cm^{-1}; (B) a partial spectrum in 400 ~ 850 cm^{-1} and (C) a partial spectrum in 900 ~ 1700 cm^{-1}.

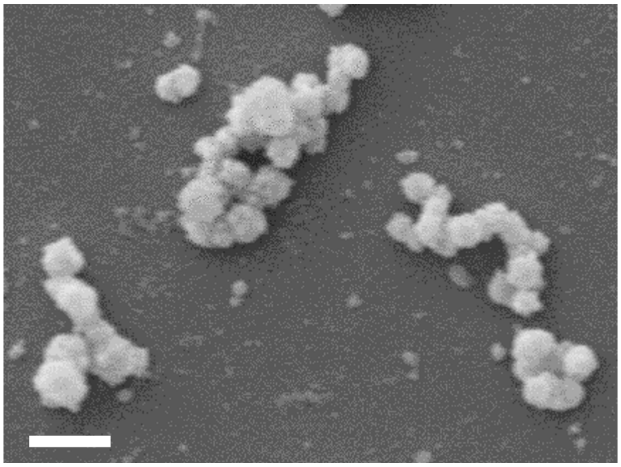

Figure S7. An SEM image of SF@MnO_2 nanoparticles obtained after the bioinspired mineralization process for 1 h (scale bar: 500 nm).

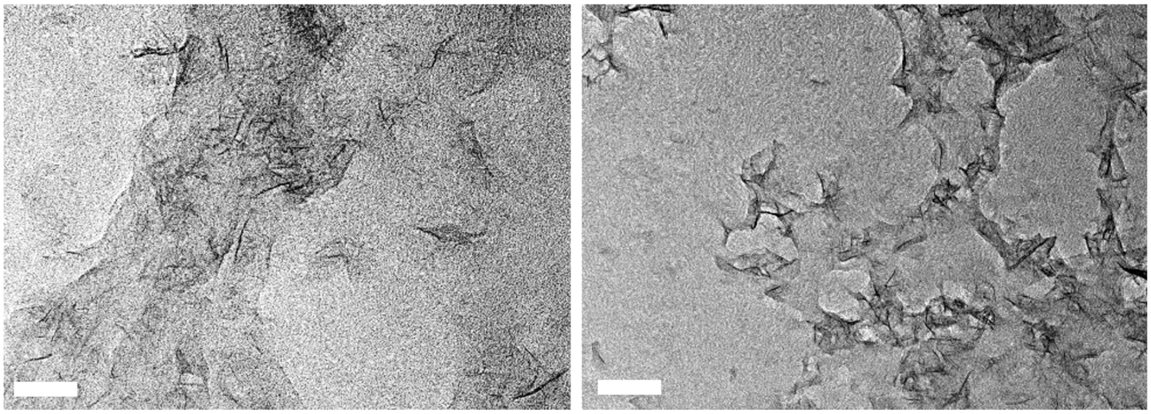

Figure S8. TEM images of SF@MnO_2 nanoparticles obtained after the bioinspired mineralization reaction for 24 h (scale bars: 100 nm).

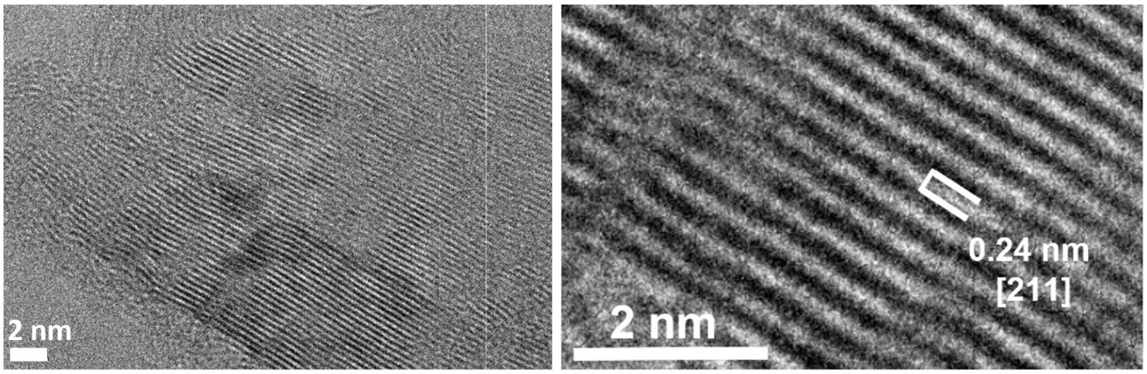

Figure S9. High-resolution TEM images of SF@MnO_2 nanoparticles under different magnifications.

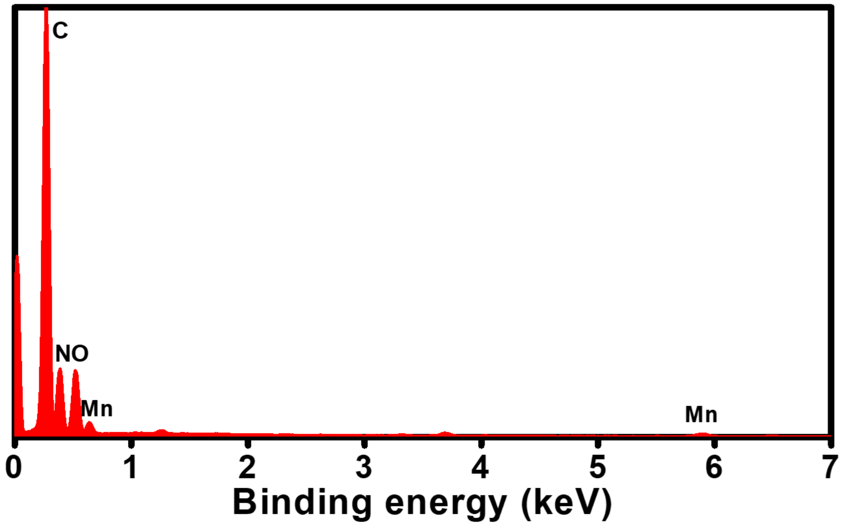

Figure S10. EDS pattern of SF@MnO_2 nanoparticles.

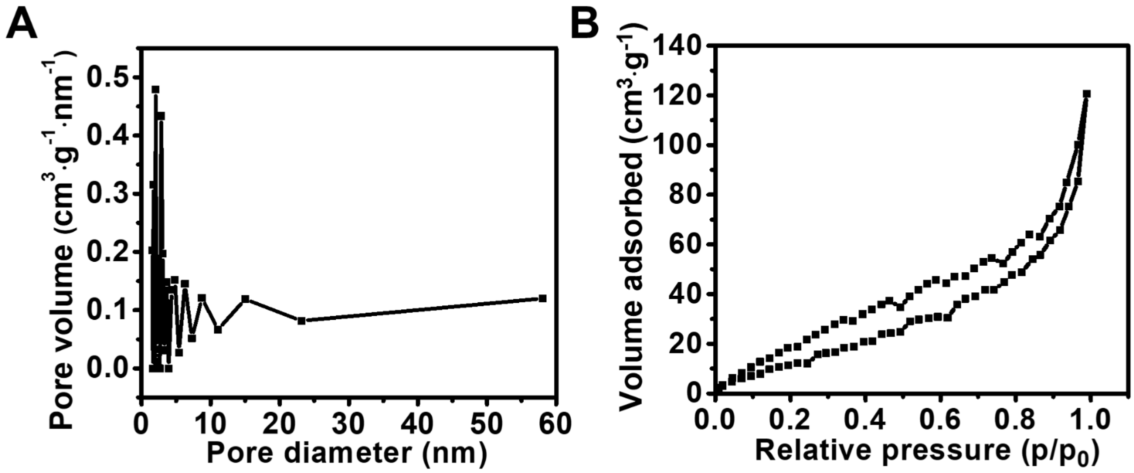

Figure S11. (A) Pore size distribution of SF@MnO_2 nanoparticles; (B) N_2 adsorption−desorption isotherm of SF@MnO_2 nanoparticles.

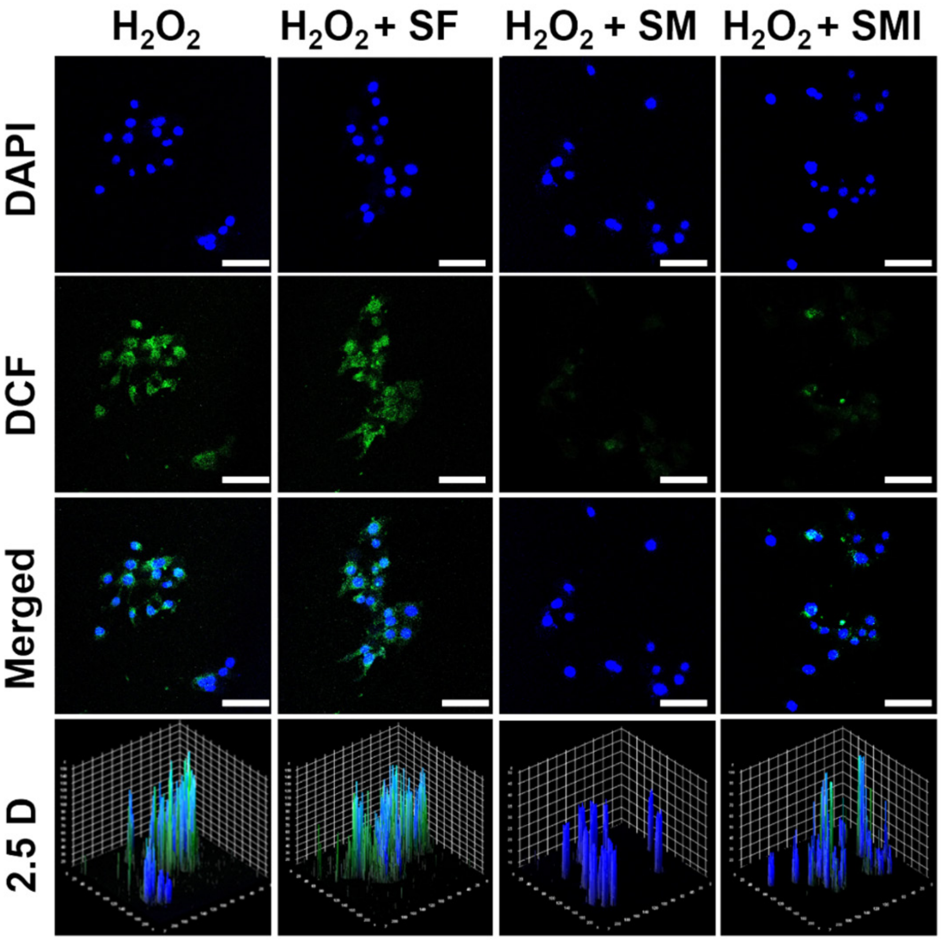

Figure S12. Fluorescence images of 4T1 cells after treatment with various agents (equivalent ICG concentration: 10 μg·mL^{-1}) in the presence of 1 mM of H_2O_2 (scale bars: 50 μm).

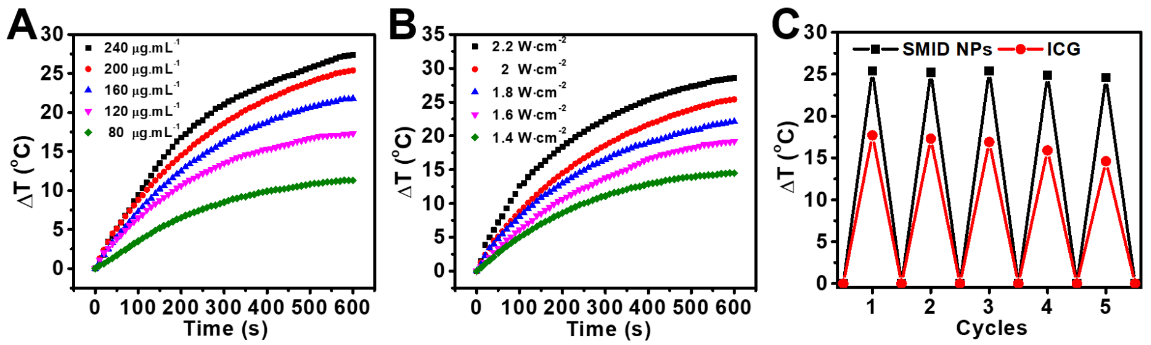

Figure S13. (A) Temperature elevation of SMID nanoparticle dispersions at various concentrations subject to NIR laser irradiation (808 nm, 2 W·cm^{-2}) for 10 min; (B) temperature elevation of SMID nanoparticle dispersions (equivalent ICG concentration: 16 µg·mL^{-1}) under exposure to NIR laser with different output power densities; (C) peak temperature of ICG and SMID nanoparticle dispersion (equivalent ICG concentration: 16 µg·mL^{-1}) subject to periodic NIR laser irradiations (808 nm, 2 W·cm^{-2}) for 5 cycles.

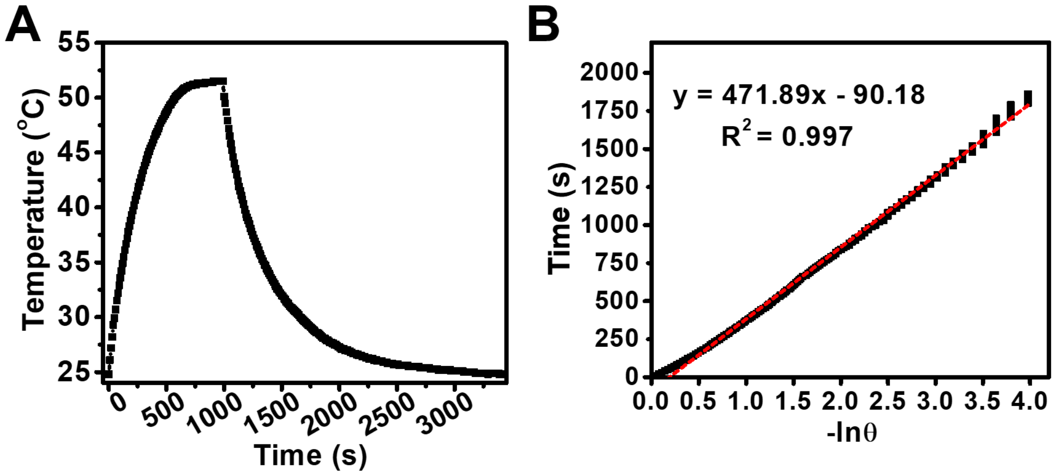

Figure S14. (A) Heating and cooling curves of SMID nanoparticle suspension (equivalent ICG concentration: 16 µg·mL^{-1}) subject to NIR laser irradiation; (B) diagram of the time versus -ln(θ) derived from the cooling stage in (C).

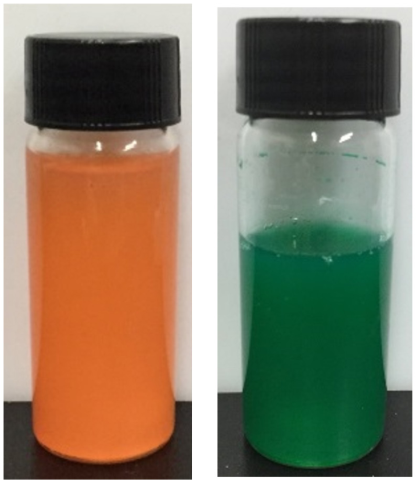

Figure S15. Images of SF nanoparticle dispersions after loading DOX (left) or ICG (right).

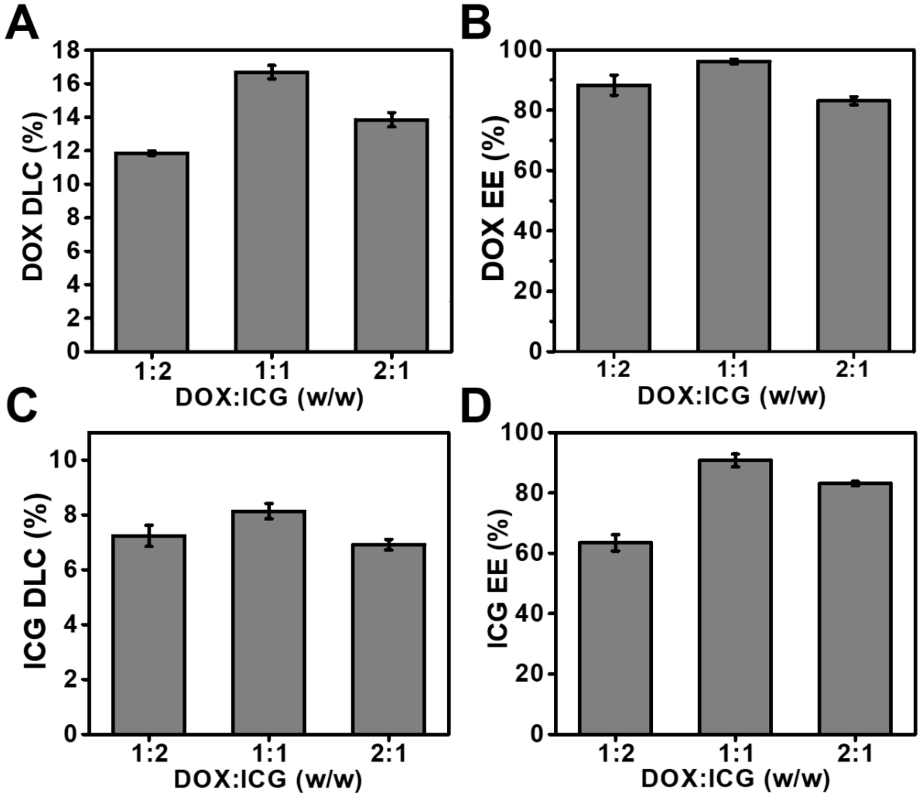

Figure S16. (A) DLC and (B) EE of DOX in SMID nanoparticles achieved under various mass ratios of fed drugs; (C) DLC and (D) EE of ICG in SMID nanoparticles achieved under various mass ratios of fed drugs.

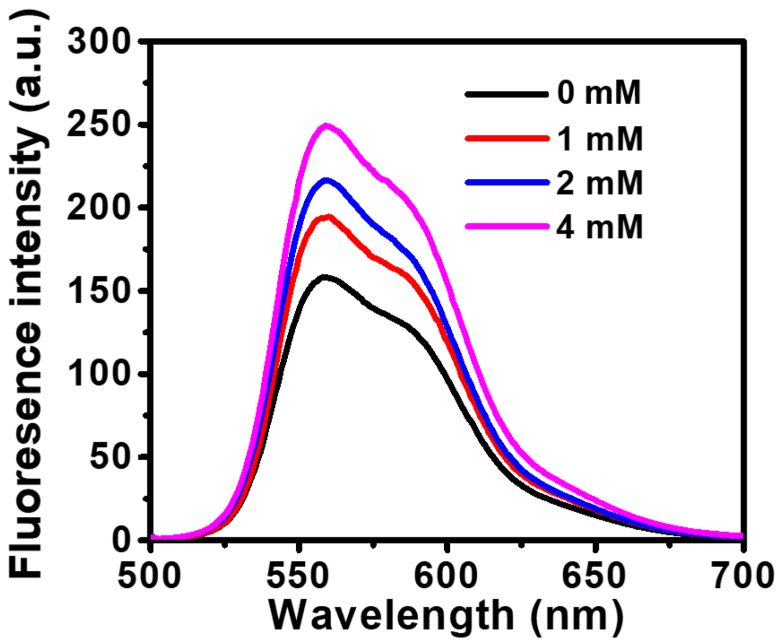

Figure S17. Fluorescence spectroscopy of different releasing media (10 mL) showing the released DOX from SMID nanoparticles (2 mg) after 30 min of incubation. Four releasing media tested: buffer solutions (pH = 7.4) containing 0 mM, 1 mM, 2 mM and 4 mM H_2O_2. Excitation wavelength: 488 nm.

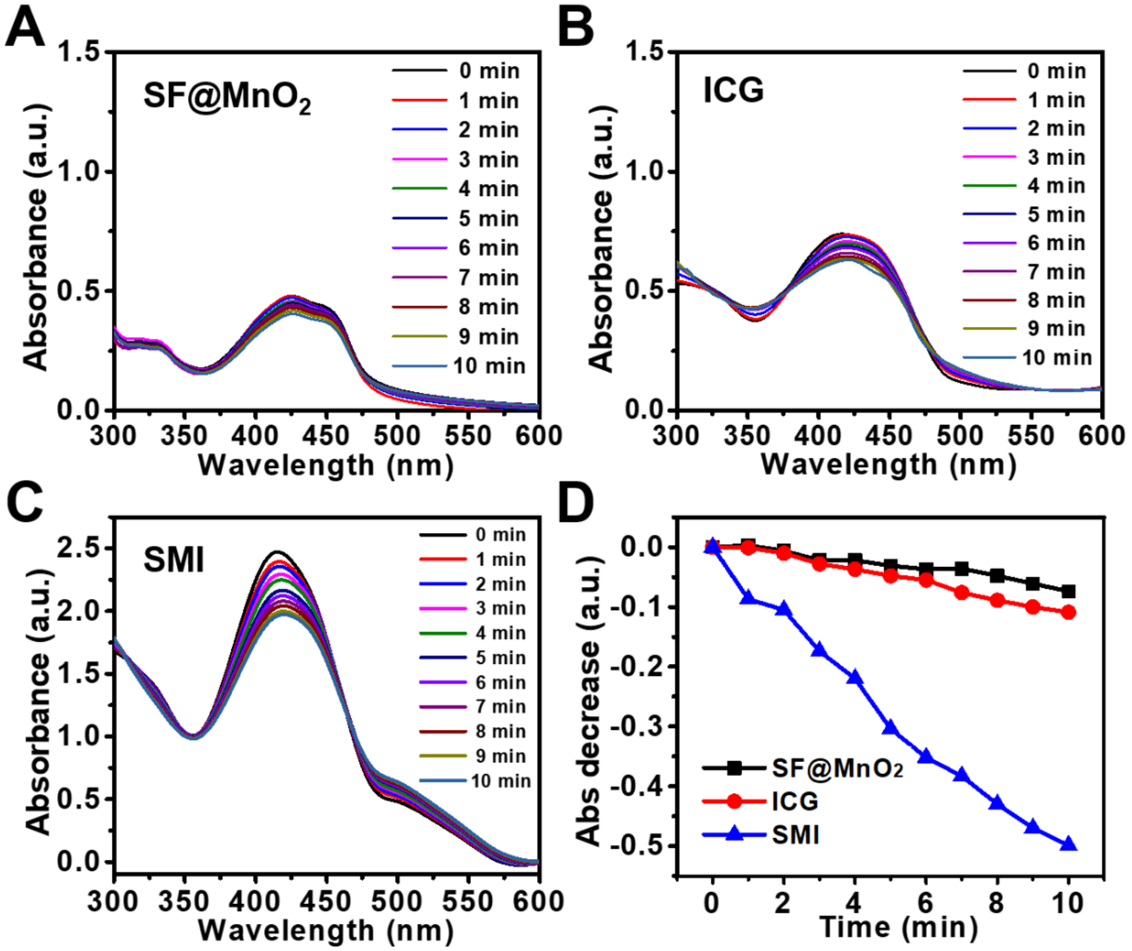

Figure S18. Optical absorbance spectra of DPBF incubated with (A) SF@MnO_2, (B) ICG and (C) SMI (equivalent ICG concentration: 10 μg·mL^{-1}) under laser irradiation (808 nm, 2 W·cm^{-2}) for various periods of time; (D) decay of the normalized peak absorbance intensity of DPBF at 417 nm as a function of irradiation time.

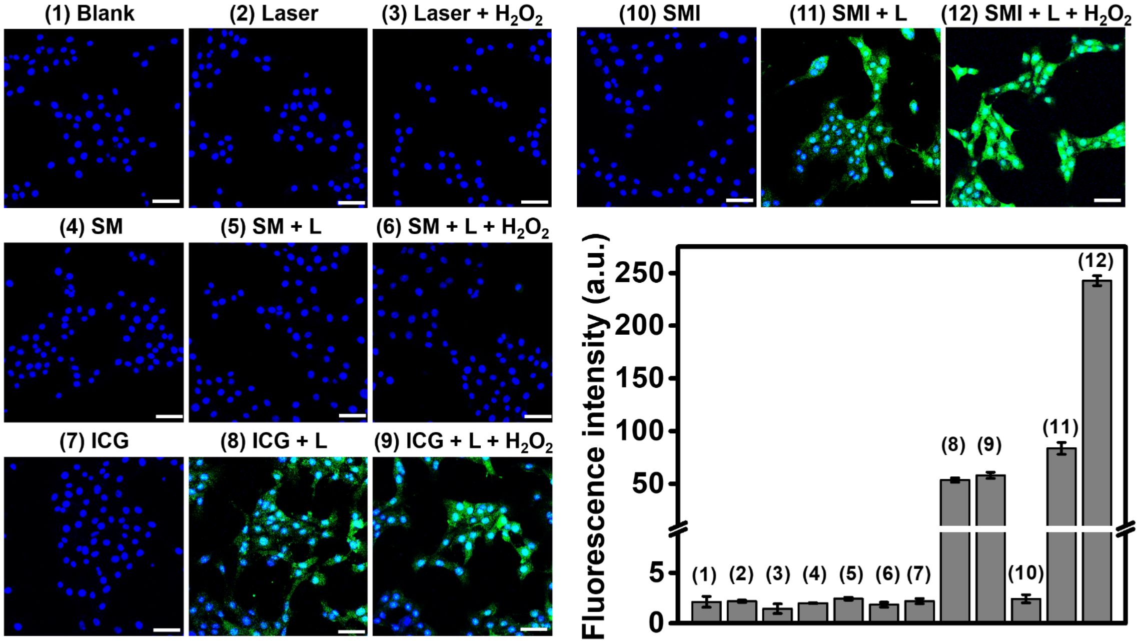

Figure S19. Fluorescence images and the corresponding fluorescence intensity (FITC channel) of SOSG-labeled 4T1 cells after treatment with various agents and NIR laser irradiation (10 min) or H_2O_2 (100 μM) where applicable. Scale bars: 50 μm.

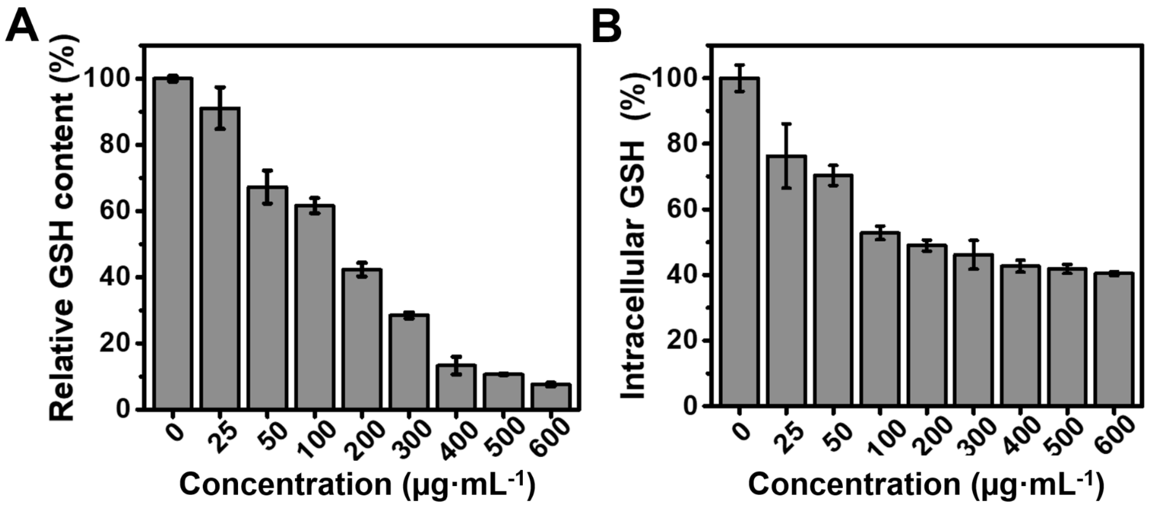

Figure S20. (A) Relative content of GSH after reaction with SF@MnO_2 at various concentrations in a test tube; (B) intracellular GSH content after treatment with SF@MnO_2 at various concentrations.

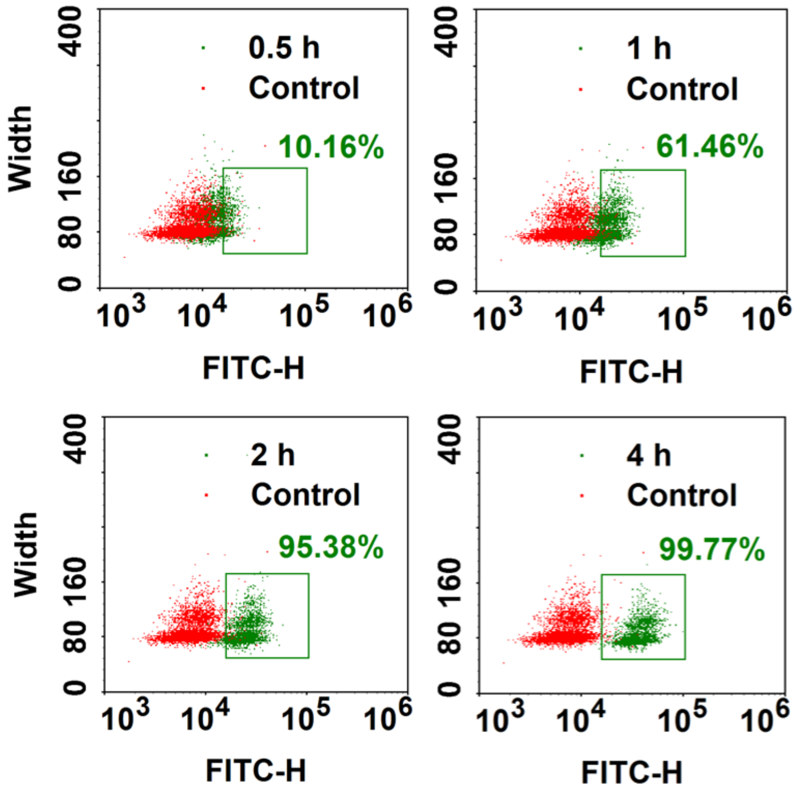

Figure S21. Flow cytometry analysis of 4T1 cells incubated with SMID nanoparticles over time (0.5 h, 1 h, 2 h and 4 h).

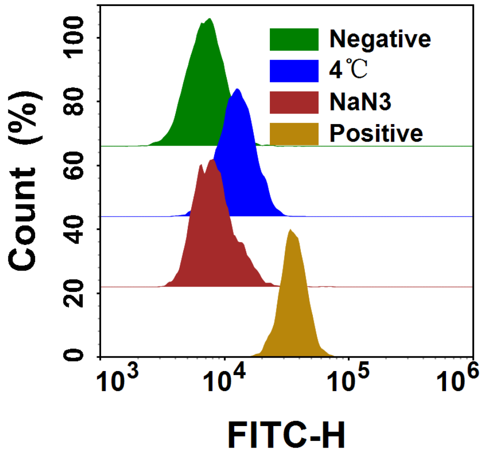

Figure S22. Flow cytometry analysis of the cellular uptake of SMID nanoparticles in 4T1 cells treated with NaN_3 (0.1%, w/v) or under 4°C for 4 h. “Positive control” denotes 4T1 cells incubated with SMID nanoparticles without any additional treatment.

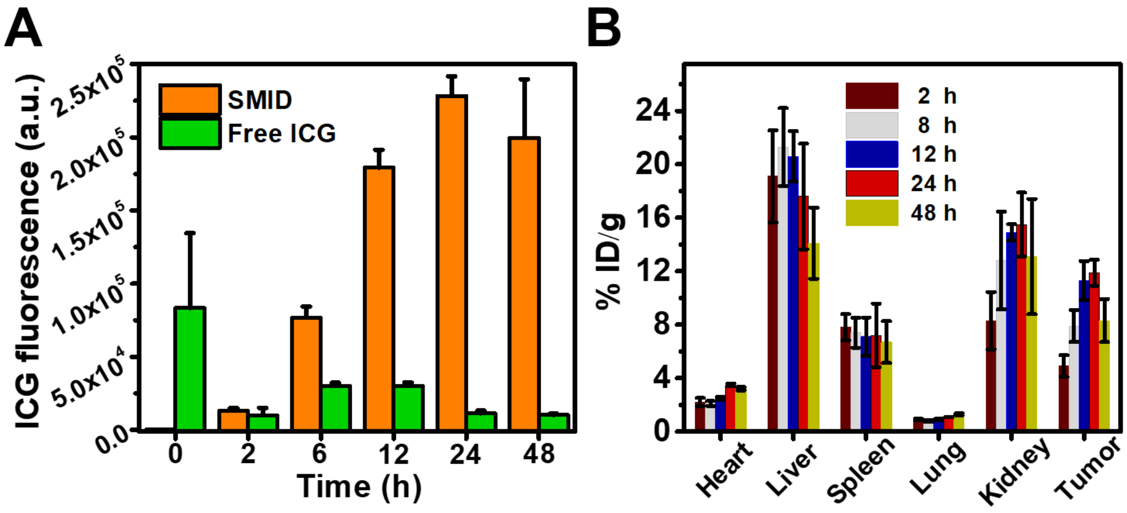

Figure S23. (A) Average fluorescence intensity of ICG in tumor region analyzed by ImageJ (v1.47) corresponding to Figure 6a; (B) biodistribution of Mn content in tumor-bearing BALB/c mice at different time points post-injection of SMID nanoparticles.

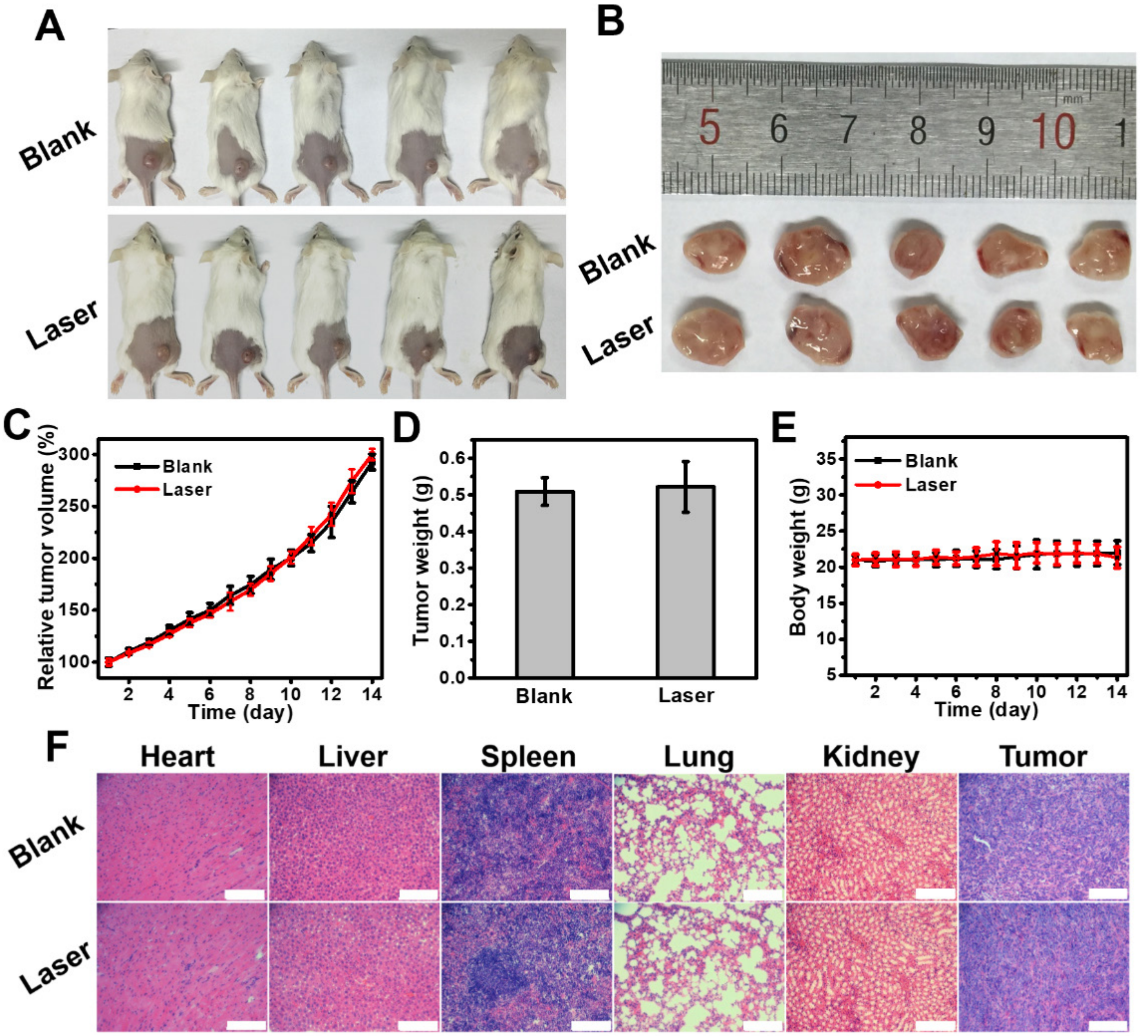

Figure S24. Evaluating the biosafety of NIR laser irradiation: (A) photographs of 4T1 tumor-bearing mice at day 14 after laser irradiation (808 nm, 2 W·cm^{-2}) or without any treatment; (B) photographs of excised tumors at day 14 after different treatments; (C) variation of relative tumor volume in 14 days after various treatments; (D) average weight of dissected tumors at day 14; (E) variation of mouse body weight in 14 days after various treatments; (F) H&E staining of histological sections sliced from tumors or major organs in different groups.

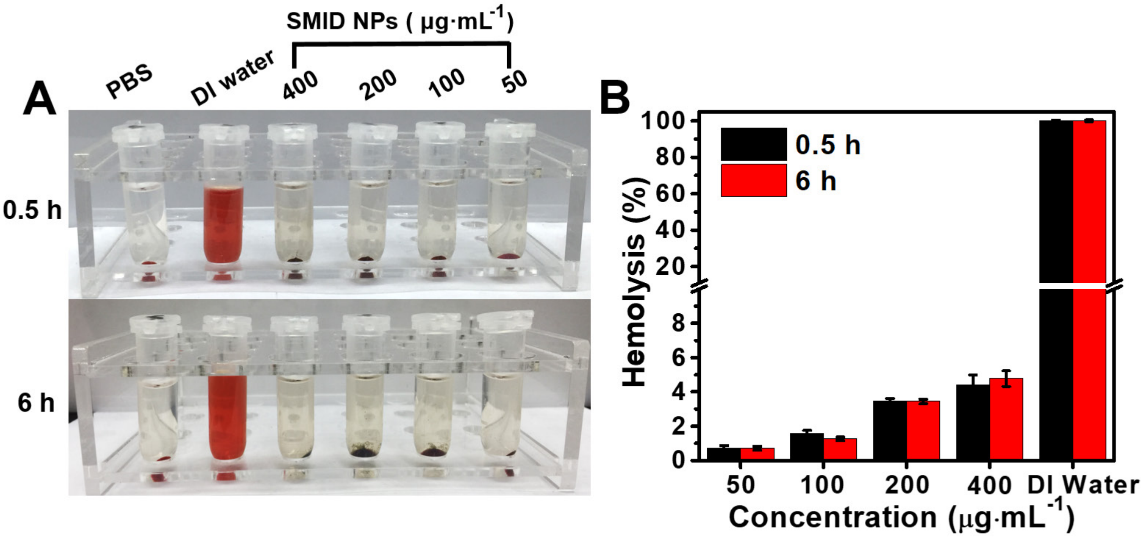

Figure S25. Hemocompatibility analysis: (A) photographs of erythrocytes incubated with SMID nanoparticles under various concentrations. Erythrocytes suspended in PBS and DI water served as negative and positive controls, respectively; (B) hemolytic rate of erythrocytes incubated with SMID nanoparticles under various concentrations.

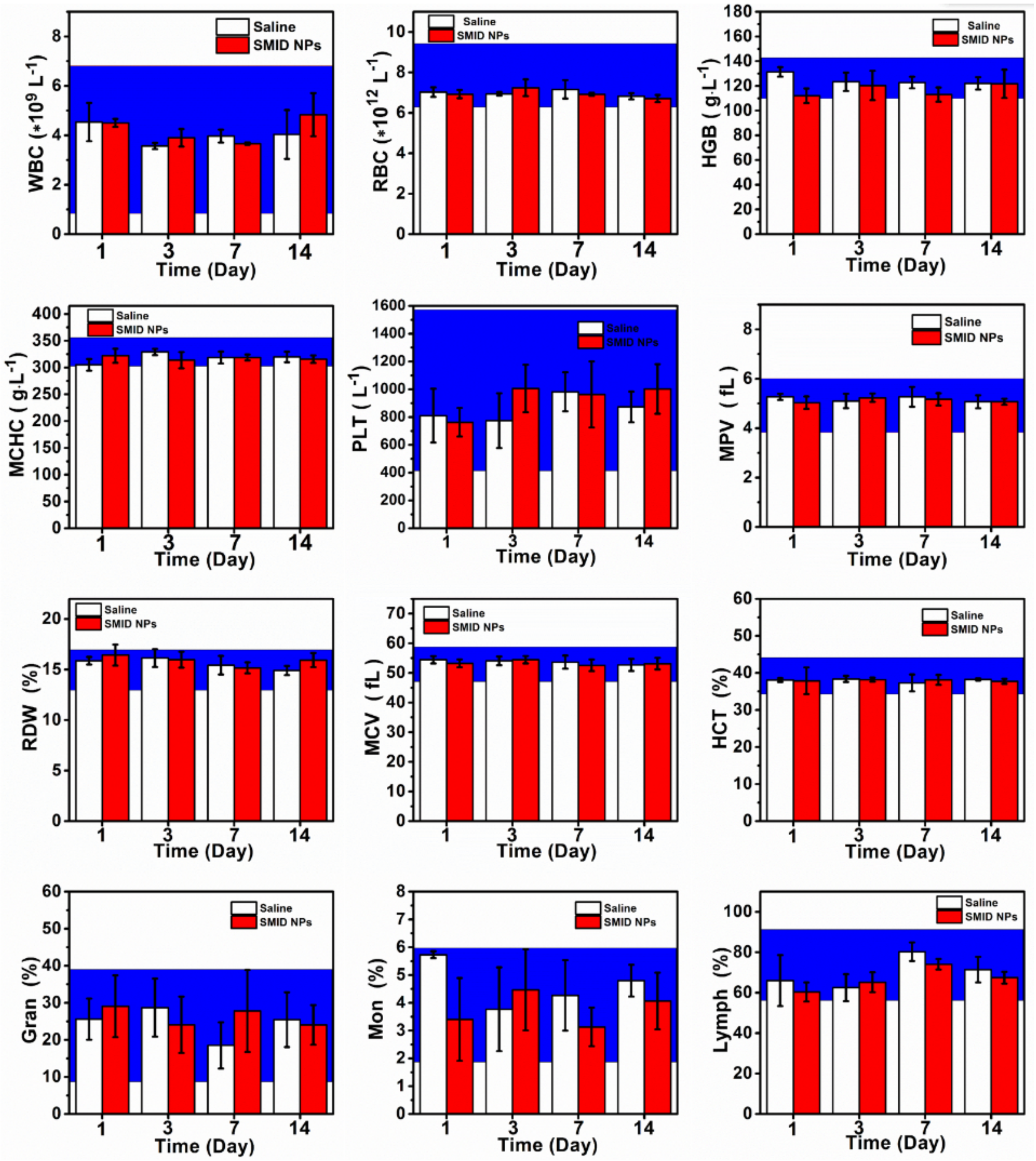

Figure S26. Complete blood counts of the mice intravenously injected with saline or SMID nanoparticles. The blue hatched areas represent the reference ranges of hematology data of healthy female KM mice.

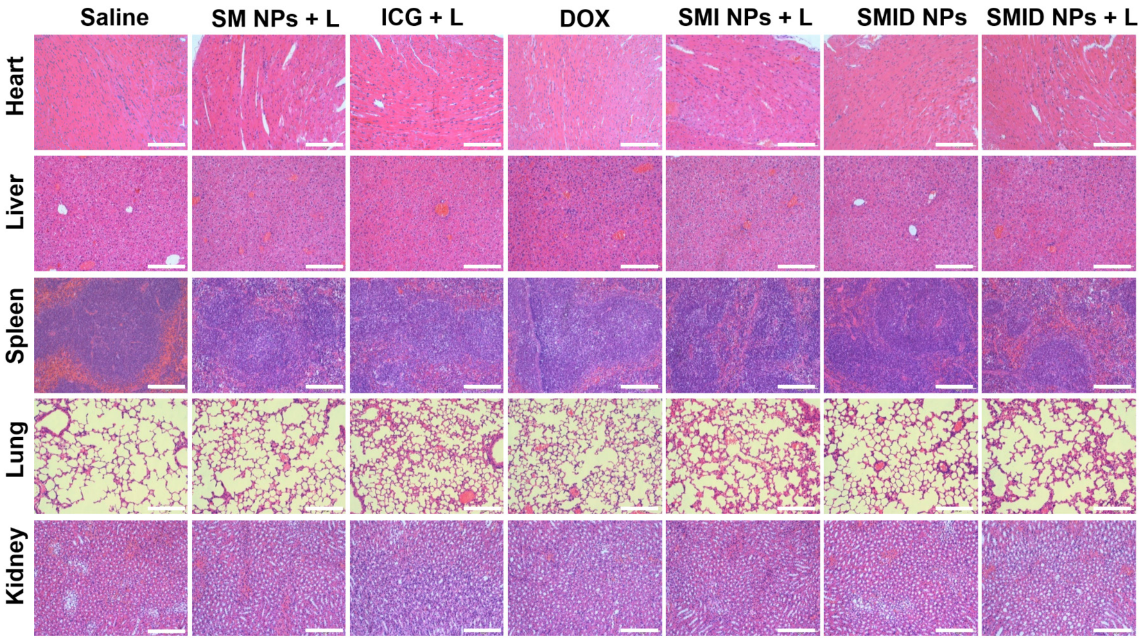

Figure S27. Histological analysis of major organs (heart, liver, spleen, lung and kidney) by H&E staining at day 14 post-injection (scale bars: 200 μm).

Supplementary Methods

Calculation of the photothermal conversion efficiency of SMID nanoparticles[1]

The total energy balance between the input and dissipation for the system is presented as:

$$\sum_i M_i C_i \frac{dT}{dt} = Q_{NP} + Q_{sys} - Q_{out} \tag{1}$$

where M and C denotes the mass and heat capacity of water, respectively; T represents the medium temperature; Q_{NP} is the energy absorbed by nanoparticles; Q_{sys} denotes the energy from the pure water system; Q_{out} is heat dissipation from the system.

The heat absorbed by SMID NPs can be calculated as:

$$Q_{NP} = I(1 - 10^{-A_{808}})\eta \tag{2}$$

where I is the power of NIR laser, η indicates the photothermal conversion efficiency, and A_{808} denotes the absorbance of SMID nanoparticles at 808 nm.

Heat dissipation is linear to the system temperature, defined as:

$$Q_{out} = hS(T - T_{surr}) \tag{3}$$

where h is the heat transfer coefficient, S is surface area of the container, and T_{surr} is the ambient temperature.

After reaching a steady state temperature (T_{max}), the input and output of heat are in equilibrium.

$$Q_{NP} + Q_{sys} = Q_{out} = hS(T_{max} - T_{surr}) \tag{4}$$

Upon removal of laser, $Q_{NP} + Q_{sys} = 0$, Eq. (1) can be converted to:

$$\sum_i M_iC_i \frac{dT}{dt} = -Q_{out} = -hS(T - T_{surr}) \tag{5}$$

$$dt = \frac{\sum_i M_iC_i}{hS}\frac{dT}{(T-T_{surr})} \tag{6}$$

$$t = -\frac{\sum_i M_iC_i}{hS}\ln\frac{T-T_{surr}}{(T_{max}-T_{surr})} \tag{7}$$

A system time constant τ_s can be defined as

$$\tau_s = -\frac{\sum_i M_iC_i}{hS} \tag{8}$$

and θ is introduced for substitution,

$$\theta = \frac{T-T_{surr}}{(T_{max}-T_{surr})} \tag{9}$$

which transforms Eq.(8) and Eq (9) into:

$$\mathrm{t} = -\tau_s \ln\theta \tag{10}$$

Since Q_{sys} can be calculated based on

$$Q_{sys} = hS(T_{max,H_2O} - T_{surr}) \tag{11}$$

Eq. (4) can be expressed as

$$Q_{NP} = I(1 - 10^{-A_{808}})\eta = hS(T_{max} - T_{max,H_2O}) \tag{12}$$

$$hS = -\frac{\sum_i M_iC_i}{\tau_s} \tag{13}$$

where τ_s is equal to 471.892 s, m is 3.0 g and c is 4.2 J/g, h_S can be calculated as 0.0267 W/^oC. Substituting I = 2.0 W, A_{808} = 4.173, $T_{max} - T_{surr}$ = 26.7 ^oC into Eq. (12), the photothermal conversion efficiency of SMID nanoparticles can be determined as 35.65%.

Reference

1. Tian, Q.; Jiang, F.; Zou, R.; Liu, Q.; Chen, Z.; Zhu, M.; Yang, S.; Wang, J.; Wang, J.; Hu, J. Hydrophilic Cu9S5 Nanocrystals: A Photothermal Agent with a 25.7% Heat Conversion Efficiency for Photothermal Ablation of Cancer Cells in Vivo. *ACS Nano* **2011**, *5*, 9761-9771.
